# Structure Based Identification and Characterization of Flavonoids That Disrupt Human Papillomavirus-16 E6 Function

**DOI:** 10.1371/journal.pone.0084506

**Published:** 2013-12-23

**Authors:** Jonathan J. Cherry, Anne Rietz, Anna Malinkevich, Yuqi Liu, Meng Xie, Matthew Bartolowits, V. Jo Davisson, James D. Baleja, Elliot J. Androphy

**Affiliations:** 1 Department of Medicine, University of Massachusetts Medical School, Worcester, Massachusetts, United States of America; 2 Department of Dermatology, Indiana University School of Medicine, Indianapolis, Indiana, United States of America; 3 Department of Biochemistry, Tufts University School of Medicine, Boston, Massachusetts, United States of America; 4 Department of Medicinal Chemistry and Molecular Pharmacology, Purdue University College of Pharmacy, West Lafayette, Indiana, United States of America; National Institute of Health - National Cancer Institute, United States of America

## Abstract

Expression and function of the human papillomavirus (HPV) early protein 6 (E6) is necessary for viral replication and oncogenesis in cervical cancers. HPV E6 targets the tumor suppressor protein p53 for degradation. To achieve this, “high-risk” HPV E6 proteins bind to and modify the target specificity of the ubiquitin ligase E6AP (E6 associated protein). This E6-dependent loss of p53 enables the virus to bypass host cell defenses and facilitates virally induced activation of the cell cycle progression during viral replication. Disruption of the interaction between E6 and E6AP and stabilization of p53 should decrease viability and proliferation of HPV positive cells. A new *in vitro* high-throughput binding assay was developed to assay binding between HPV-16 E6 and E6AP and to identify compounds that inhibit this interaction. The compound luteolin emerged from the screen and a library of novel flavones based on its structure was synthesized and characterized using this *in vitro* binding assay. The compounds identified in this study disrupt the E6/E6AP interaction, increase the levels of p53 and p21^Cip1/Waf1^, and decrease proliferation of HPV positive cell lines. The new class of flavonoid E6 inhibitors displays a high degree of specificity for HPV positive cells. Docking analyses suggest that these compounds bind in a hydrophobic pocket at the interface between E6 and E6AP and mimic the leucines in the conserved α-helical motif of E6AP. The activity and specificity of these compounds represent a promising new lead for development as an antiviral therapy in the treatment of HPV infection and cervical cancer.

## Introduction

HPV causes common cutaneous, mucosal, anogenital, and oropharyngeal epithelial growths. Genital warts are highly transmissible and affect all socioeconomic groups. The CDC estimated there are ~750,000 new cases of genital warts each year and 1.5 million persons under treatment in the USA. Annually three million new cases of abnormal Pap smears are detected in the USA, indicating active HPV infection. A minority of these lesions progress to pre-cancerous dysplasia and to invasive malignancy. On a worldwide basis, ~500,000 new cases of cervical cancer are diagnosed and nearly 250,000 deaths occur each year. HPV type 16 is found in approximately 50% of all cervical cancers [1] and is the most frequent isolate from oropharyngeal cancers, of which 25-50% are attributed to HPV [2–4].

The HPV-E6 protein is essential for viral replication and instrumental in bypassing host cell defenses and preventing apoptosis [5–7]. The best-known function of HPV E6 is its ability to target the tumor suppressor p53 for degradation. The cervical cancer associated or “high-risk” HPV-E6 proteins directly bind the ubiquitin ligase E6AP and targets p53 for inactivation by inducing its degradation at the proteasome [8–10]. p53 regulates cell growth and is the most commonly mutated tumor suppressor gene in human malignancies [11,12]. The E6 proteins from high-risk viruses are similar in amino acid sequence, bind E6AP, and degrade p53. High-risk HPV genomes with mutations in E6 that prevent p53 degradation do not replicate in primary keratinocytes [13,14]. E6 binds to a conserved α-helical motif found in E6AP and several other cellular factors [5,6,15–18]. E6 can also increase telomerase activity and forestall replicative senescence [19,20]. Its C-terminal region binds to members of PDZ domain family of proteins including hDlg, MAGI, and scribble [21,22]; this region is not required for its interaction with or degradation of p53 [23–25]. 

High-risk E6 and E7 together efficiently immortalize primary human keratinocytes [26–28] and E6 alone immortalizes human mammary epithelial cells [29]. E7 binds to the retinoblastoma protein (pRb), disrupts cell cycle control, and inactivates this tumor suppressor pathway [30,31]. Transgenic mice have been used to dissect the roles of these genes during tumorigenesis. While E7 was found to be involved in promoting tumor formation, E6 plays a major role in tumor progression [32]. 

Several cellular models show that continued expression of E6 is necessary to maintain the transformed phenotype. Over-expression of papillomavirus E2 protein represses expression of E6 and E7 and induces HeLa cell senescence [33–35]. Decreased expression of E6 mediated by RNAi results in growth arrest, senescence, and in some cases apoptotic cell death of several HPV positive cervical cancer cell lines [36,37]. Because these activities are essential features of HPV-induced infection and oncogenesis, inhibition of E6 function is an ideal target for an anti-viral drug.

Using our previous pharmacophore for the E6AP charged leucine helical motif [18], a new *in silico* screen was performed to identify a novel series of compounds that can inhibit the interaction between HPV-16 E6 and E6AP. A selection of naturally occurring flavonoid analogs displayed the best inhibitory activity and highest potency. We describe the activity of two compounds: the naturally occurring flavonoid luteolin and the novel flavone analog CAF-24. Both displayed a low micromolar IC_50_ in our *in vitro* binding assay, elicited a potent increase in p53 and p21^Cip1/Waf1^ protein, and decreased viability of HPV positive cell lines. We present evidence that luteolin can interact directly with HPV-16 E6. Docking analysis using the recently resolved structure of HPV16 E6 [38] suggests that these compounds bind in a hydrophobic pocket at the interface between E6 and E6AP. 

## Results

### Filter plate based HPV-16 E6/E6AP interaction assay for lead conformation

To characterize potential E6 inhibitors, a robust *in vitro* assay was developed to measure the interaction between HPV-16 E6 and E6AP. Although results from this assay have been published previously [18,39], this is the first time we have describe its development in detail. The core 70 amino acids of the E6 binding region of E6AP (residues 371-440) [8] was fused to bacterial alkaline phosphatase (BAP) in the MY101 vector [40]. In preliminary experiments, this fusion was more stable and bound with higher affinity than a fusion of BAP with the 18 amino acid α-helical core E6 binding motif in E6AP (data not shown). Briefly, glutathione-bead bound GST-16 E6 was immobilized on 96-well filter plates, incubated with free E6AP-BAP fusion protein, and unbound E6AP-BAP fusion was washed from the plates. Binding efficiency was determined from the intensity of BAP activated chemiluminescence remaining in each well. 

To calculate the signal to background ratio, binding was tested in the presence of GST-E6, GST alone, or in empty wells. The signal from the E6AP-BAP fusion was 20-fold greater in the wells with GST-E6 than in empty wells or those containing GST alone. Binding was subsequently examined in the presence of DMSO, the solvent present in the library, and up to 20% DMSO was tolerated without loss of signal (data not shown). 

**Figure 1:**
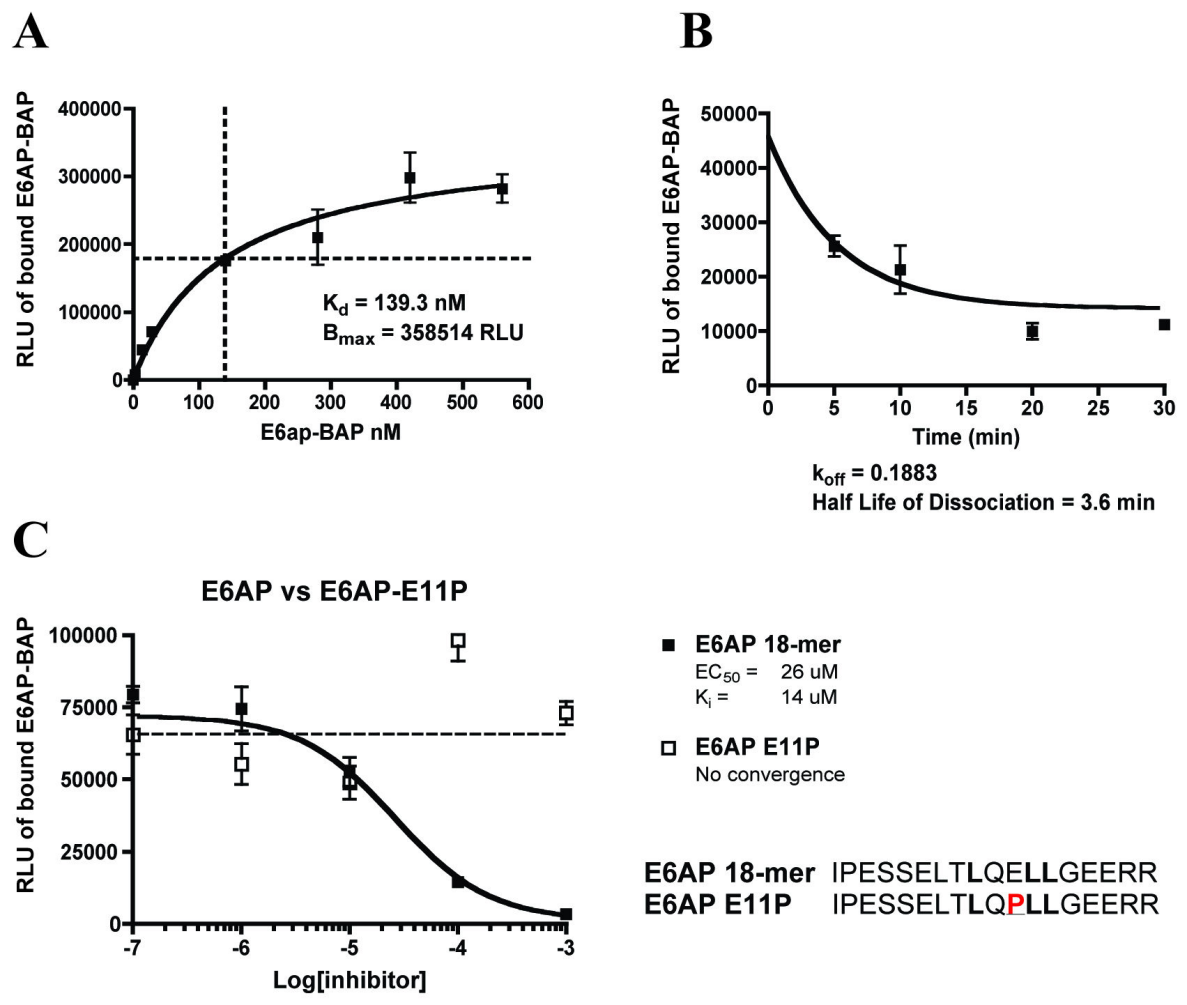
Kinetic Characterization of the *in vitro* GST-E6/E6AP-BAP Binding Assay. (**A**) Saturation binding assay with GST-E6 concentration at 500 ng per well (400 nM) while E6AP-BAP concentration was increased. (**B**) Dissociation rate experiment. GST-E6 and E6AP-BAP were allowed to bind to equilibrium and unbound E6AP-BAP was washed from the wells. Dissociation was measured as a decrease in BAP signal at each time point. (**C**) The E6AP 18-mer peptide inhibits the interaction between E6AP-BAP and GST-16 E6. E11P peptide is unable to efficiently bind E6 and cannot compete with E6AP-BAP.

Kinetic analysis was performed to characterize the protein-protein interaction inherent in this assay. The equilibrium dissociation constant (K_d_) of 139 nM was ascertained by a saturation binding experiment (Figure **1A**). This was more than 20-fold lower (i.e. tighter binding) than the value reported for a smaller E6AP fusion that contains residues 403–417 [41]. The off-rate (k_off_) was determined in a dissociation binding experiment as 0.19 min^-1^ and the half-life of ligand dissociation (t_1/2_) was calculated to be 3.6 minutes (Figure **1B**). This information confirmed that 1 hour incubations were sufficient for the competitive binding assays to reach saturation. 

To assess inhibition of this protein-protein interaction, an 18-mer peptide derived from E6AP was used as a positive control (Figure **1C**) that corresponds to the α-helical leucine motif necessary for the interaction between E6 and E6AP [15,42]. This peptide folds correctly [16], interacts with purified E6 by NMR [43] and can directly compete with and disrupt the binding between GST-E6 and E6AP-BAP. The E6AP proline mutant E11P 18-mer was used as a negative control (Figure **1C**) [15,16]. This peptide has a proline substitution at the core of the helical motif at position 11 that disrupts correct folding in the peptide and does not interact with E6. The E6AP 18-mer was an effective inhibitor of the interaction between E6 and E6AP-BAP with an IC_50_ of 26 µM and inhibited binding by 95% (16.6 fold) at 100 µM. The % deviation in the wells with the DMSO control and E6AP peptide was 13% and 17%, respectively. 

By comparing the well-to-well variation with the magnitude of change between control-treated and untreated cells, it is possible to statistically determine a z’-factor for the assay. The z’-factor mathematically assesses whether the changes in signal intensity are significant and if the assay can accurately distinguish “hits” from a large number of test compounds. An assay with a z’-factor > 0.5 is appropriate for high throughput screening (HTS). The z’-factor calculated for our test plates was 0.53 with a signal to noise ratio of nearly 20:1 and can be considered well suited for high content screening.

### New *in silico* screen using a pharmacophore for the E6 interaction domain

**Figure 2:**
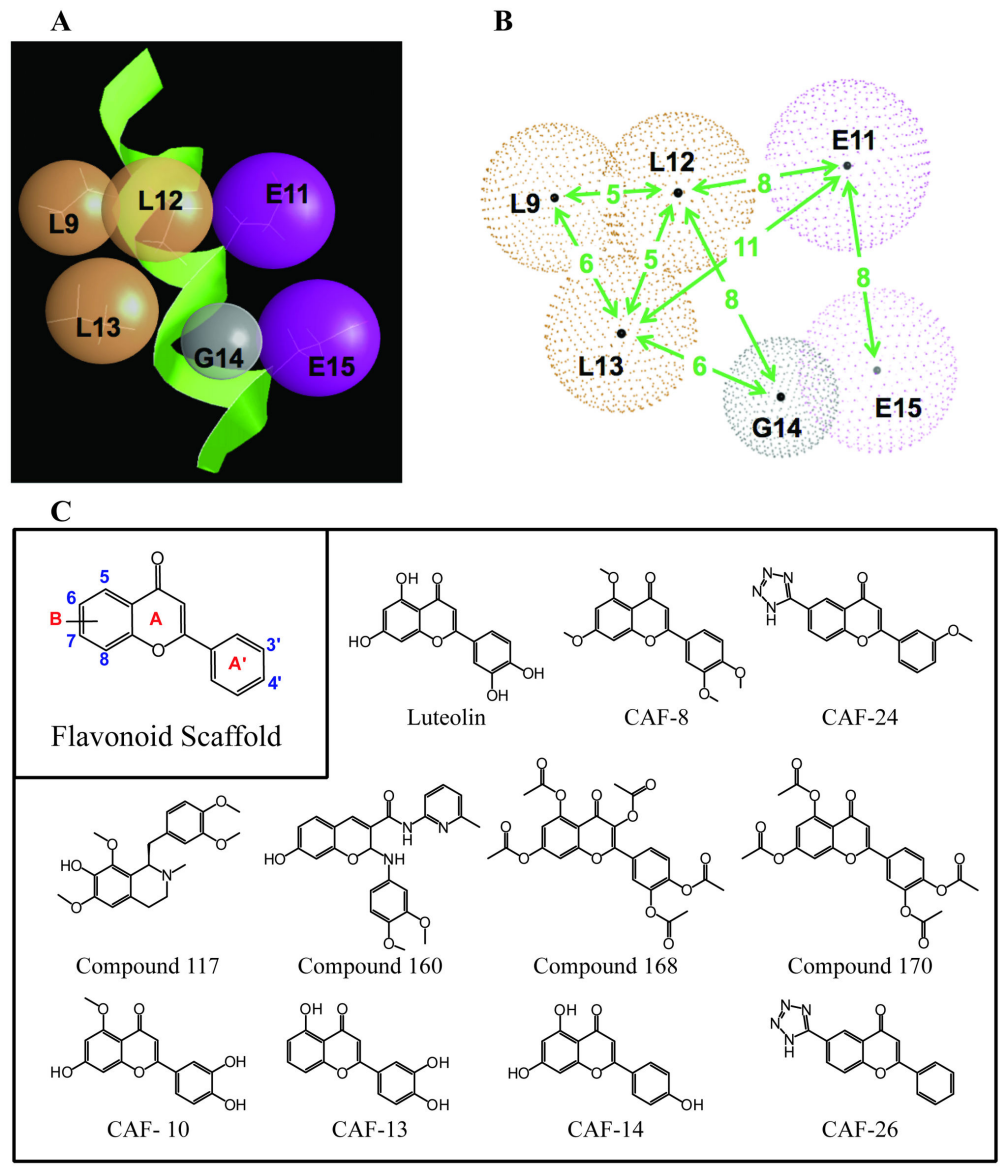
Pharmacophore design and novel flavonoid inhibitors. (**A**) The pharmacophore is mapped on the three-dimensional structure of the E6AP helical motif (PDB ID: 1EQX). The green α-helix corresponds to 18 amino acids of E6AP peptide. The residues L9, E11, L12, L13, G14, and E15 are displayed. The orange hydrophilic spheres are superimposed over the leucines at positions 9, 12, and 13. The charged spheres for positions 11 and 15 are presented in purple. The zone of exclusion around glycine 14 is represented by the gray sphere. (**B**) The distances between reactive groups were determined by the peptide structure of E6AP. Ångström distances are listed in green. The initial radius of each sphere was calculated from the ensemble of structures of the peptide. (**C**) Relevant compounds are listed. These include active compounds from the initial screen and novel flavonoid derivatives synthesized for this project.

We previously defined a six-point pharmacophore model for the identification of compounds that bind to E6 [18]. It was based on mutagenesis and three-dimensional data on LxxLL E6-interacting helical motif, including the E6AP sequence **LQELL**GE (L9-Q10-E11-L12-L13-G14-E15) [15,16]. This motif forms a helical domain with a hydrophilic surface that consists of three leucines (Figure **2A**; orange spheres) on one side of the helix and charged surface on the other that includes the 2 glutamic acid residues (Figure **2A**; purple spheres). In addition, a zone of exclusion was defined to represent the lack of reactive potential of glycine in position 14 (Figure **2A**; gray sphere).

For this study, four of the six points were combined systematically to query the National Cancer Institutes (NCI) open chemical and Synthline databases. The most successful query included the two hydrophilic spheres at L12 and L13, the hydrogen-binding sphere at E11, and the sphere of exclusion at G14. Through a pharmacophore validation step, the precise distances between each of the points and the contact radius of each sphere was determined of the pharmacophore (Figure **2B**) and adjusted iteratively to obtain a reasonable number of virtual hits from the chemical collections (251 hits of roughly 250,000) (Figure **2B**). The 251 hits were then used to perform atom-atom similarity hits from a virtual library of 4.5 million commercially available compounds to yield a set of 18,000 candidate molecules. Stochastic conformational analysis of the 18,000 molecules resulted in roughly 700,000 energetically reasonable conformations for query using the pharmacophore. Of the 714 compounds that best matched the pharmacophore, 72 were chosen for further evaluation based on expected chemical stability, molecular weight, log P and log D scores, price, and availability.

### Confirmation of *in silico* leads

The collection of 72 available structural-lead compounds was tested in the filter-plate based *in vitro* E6-E6AP binding assay. Compounds were tested twice at concentrations of 10, 50, and 100 µM. Of these, 26 compounds inhibited the E6-E6AP interaction by greater than 50%. The ten most active (inhibition >75%) and reproducible inhibitors were retested in duplicate using the *in vitro* binding assay at 5 concentrations ranging between 5 to 250 µM. To confirm specificity, these compounds were also assayed for the ability to disrupt either the interaction between anti-FLAG antibody and the FLAG epitope in the E6AP-BAP fusion. Compounds that disrupted the Flag-BAP interaction with the same potency as the HPV-16 E6 and E6AP interaction were classified as non-specific and eliminated from further analysis.

**Figure 3:**
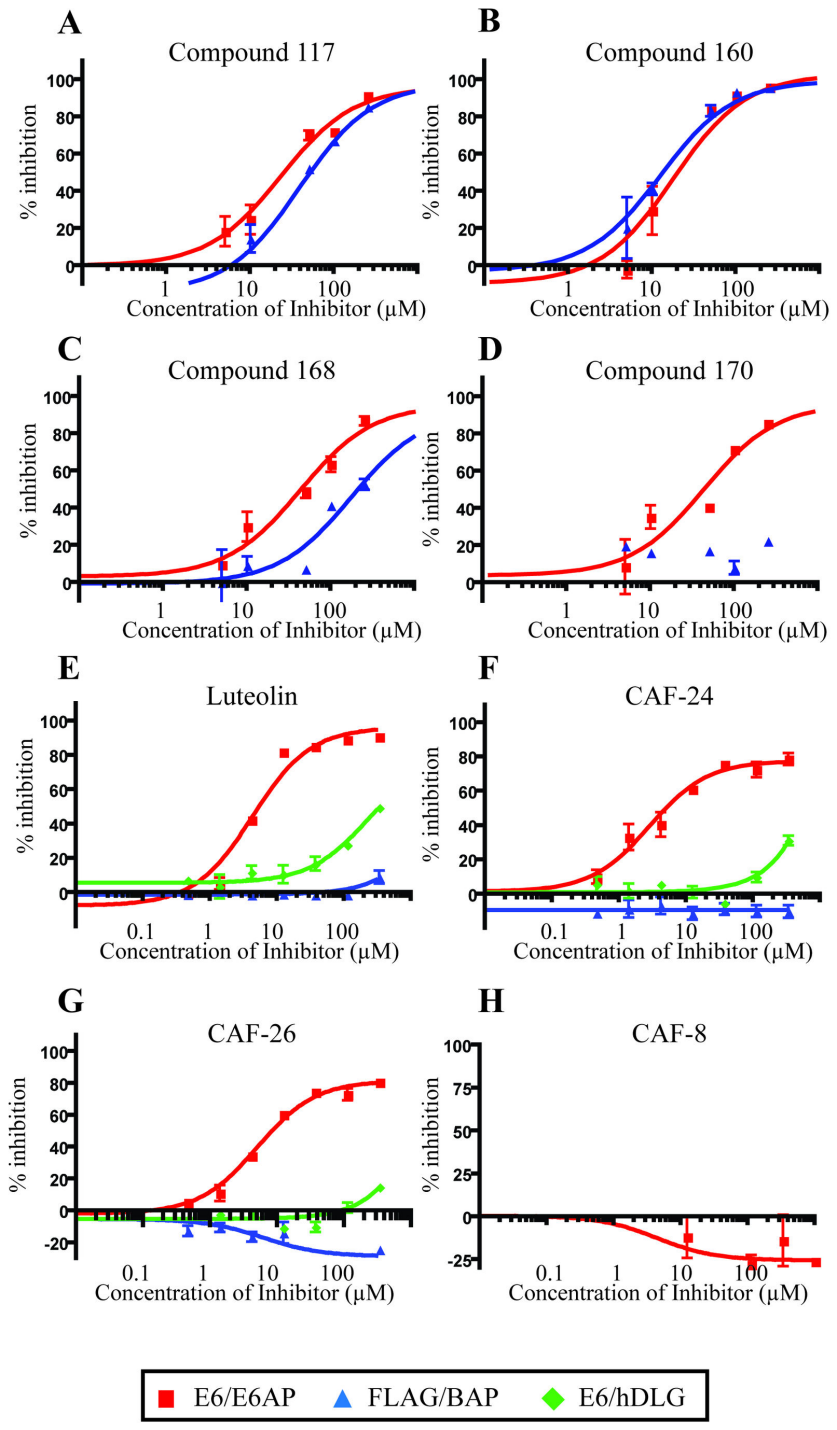
Activity of flavonoids in the *in vitro* binding assay. Compounds were tested *in* *vitro* for the ability to disrupt the E6-E6AP interaction. Increasing amounts of compound was mixed with purified GST-HPV-16 E6 and FLAG-tagged E6AP-BAP (red), HPV-16 E6 and FLAG-tagged hDlg-BAP (green), or FLAG antibody beads and FLAG-tagged E6AP-BAP (blue). Percent inhibition was determined by the amount of BAP activity remaining in the well after washing and normalized to DMSO. Data was plotted by linear regression for single site binding using Prism (n=3, S.E.M.).

The inhibitory potential of these compounds was confirmed and the IC_50_ for each calculated. Of the ten compounds tested, six did not produce a robust dose response in the E6-E6AP binding assay. The four remaining compounds; *117, 160, 168*, and *170*; did produce dose response curves with low micromolar IC_50_ values (Figure **2C** and **3**). However, *117* and *160* also inhibited binding in the FLAG-E6AP assay and were classified as non-specific inhibitors. The two remaining compounds, *168* and *170*, had a similar flavone scaffold and inhibited the E6/E6AP interaction with an IC_50_ value of 44 µM (Figure **3C,D**). These compounds are the acetylated pro-drug analogs of the naturally occurring flavonoids quercetin and luteolin (Figure **2C**). Compound *168* displayed some non-specific inhibitory activity in the FLAG-E6AP. Compound *170* was the more selective inhibitor of the E6-E6AP interaction. 

### SAR from target-library of synthesized flavone compounds

The 5,7-dihydroxy-4H-chromen-4-one core found in compounds *168* and *170* was chosen as the lead chemotype for initial structure activity relationship studies. A library of nearly 70 additional flavonoid compounds was synthesized to explore the chemical space around this scaffold and identify more potent inhibitors of the E6-E6AP interaction. All new compounds were first assayed in duplicate for inhibition of the interaction between E6 and E6AP across a 5-point dose range between 12.3 µM and 1 mM. Compounds were considered active if they inhibited the interaction between E6 and E6AP by at least 80% at a concentration equal to or less than 100 µM. Active compounds were retested in a second round of filter binding assays including the E6/E6AP-BAP assay, the FLAG/BAP assay, and an additional specificity assay that measures the interaction between GST-E6 and a fusion of three PDZ domains of hDlg onto BAP (hDlg-BAP). This interaction between E6 and hDlg occurs at the C-terminus of E6 and is independent of the interaction between E6 and the charged leucine helix in E6AP [21,22]. Compounds that directly disrupt the interaction between E6 and E6AP-BAP should not disrupt the interaction between E6 and hDlg-BAP, unless these disrupt the folding and overall conformation of the E6 protein. For this series, compounds were tested in quadruplicate at 7 concentrations ranging from 0.5 µM to 333 µM and were classified by their inhibitory activity, potency and specificity.

In this library, the 5,7-dihydroxy-4H-chromen-4-one core was held constant (Figure **2C**). Upon re-synthesis, compound *170* displayed much lower activity and potency than observed with the initial hit. However, systematic deacetylation of compound *170* revealed that the flavone luteolin was more active and more potent, with up to 100% inhibition of the interaction between E6 and E6AP and an IC_50_ of 4.3 µM in the full 7-point binding assay (Figure **3E**). Further analysis revealed that modification of the free hydroxyl groups in luteolin, including either methylation or acetylation, decreased both potency and inhibition (Table **S2**). The fully methylated analog of luteolin, CAF-8, demonstrated no activity in the initial binding assay (Figure **3H**). The one exception was methylation of the hydroxyl group at position 5 in CAF-10 (Figure **2C**, Table **S2**), which displayed the same potency and activity as luteolin. Removal of the hydroxyl groups at any position also resulted in progressive loss of activity (Table **S3**). CAF-13 and CAF-14 (Figure **2C**, Table **S3**), which lack hydroxyl groups in the 7 and 3’ positions respectively, produced a similar level of inhibition but their IC_50_ increased by 15-30 fold. 

### Identification of novel flavones with E6 inhibitor activity

A second branch of the medicinal chemistry effort focused on 4H-chromen-4-one as the core scaffold. A library of 160 compounds was generated without the hydroxyls on positions 5 and 7. As with the previous library, these compounds were initially screened for the ability to inhibit the E6-E6AP interaction by at least 80% at a concentration of 100 µM or less. Active compounds were re-screened in the E6/E6AP, FLAG/BAP, and E6/hDlg binding assays.

**Figure 4:**
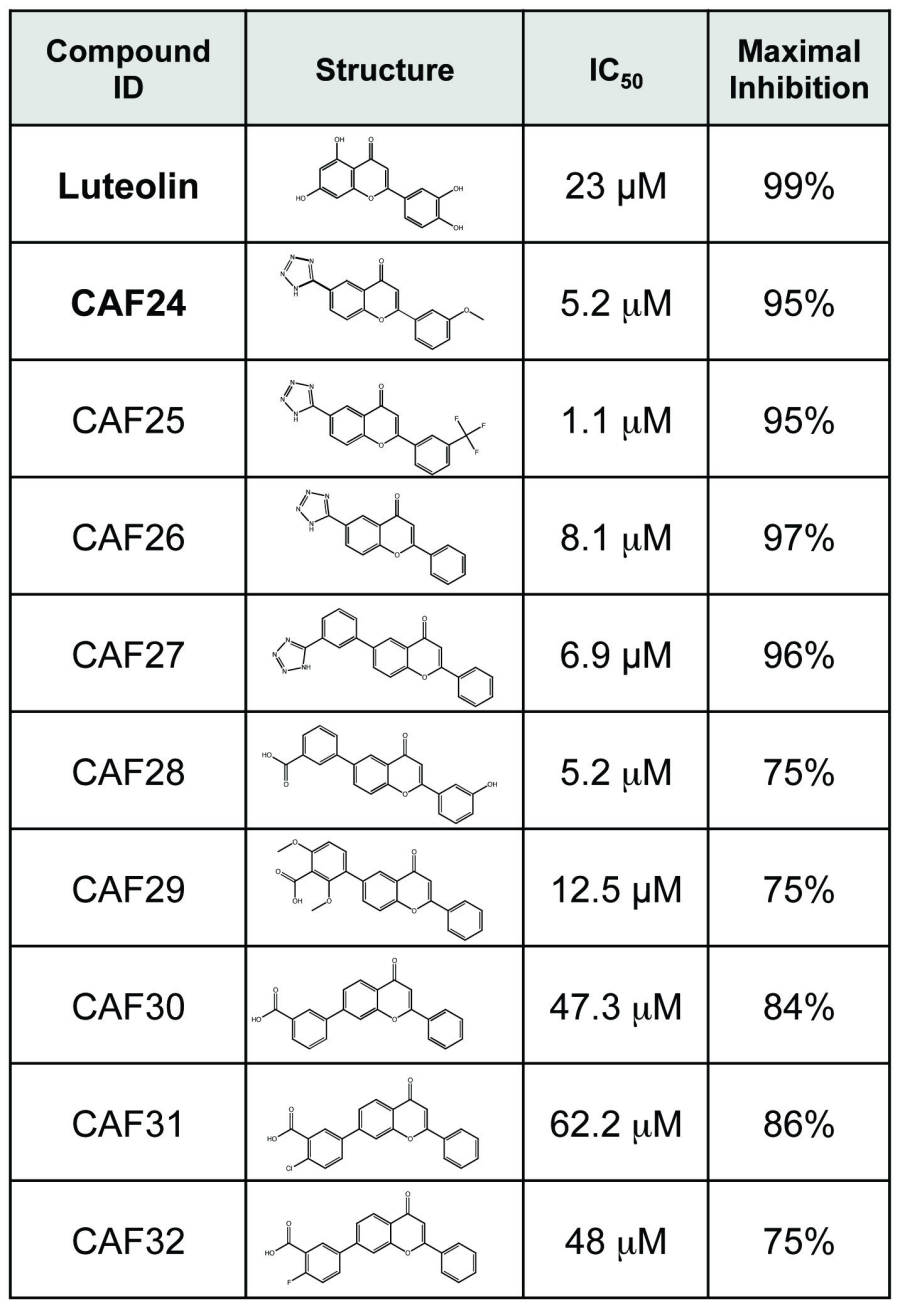
Structure activity relationship of novel flavonoid compounds. The table presents the potency and maximum inhibition of the novel flavonoid compounds in the *in* *vitro* E6/E6AP filter binding assay. These compounds lack the hydroxyl groups found on the B-ring of luteolin. These groups are replaced by a substituted benzene or heterocyclic B-ring at positions 6 or 7 to reconstitute activity.

As discussed above, masking or removal of the hydroxyls in positions 5 and 7 resulted in loss of inhibition in the *in vitro* assay (Tables **S1**-**S3**). This was likely due to loss of hydrogen bonding potential in this portion of the molecule. Addition of a substituted benzene or heterocyclic B-ring at positions 6 or 7 restored activity (Figure **4**). The B-rings in active compounds included tetrazole, benzoic acid, pthalic acid, and carboxylic acid. The addition of the B-ring supported similar levels of activity and potency to luteolin, even in the absence of the exposed hydroxyls at positions 3’ and 4’ as highlighted in the SAR for luteolin. These compounds also displayed a higher selectivity for inhibition of the E6/E6AP interaction than the controls. Two of these compounds, CAF-24 and CAF-26, had IC_50_ of 2.8 µM and 5.1 µM in the full dose response curves. Each contained a tetrazole group at position 6 and displayed activity similar to luteolin in the *in vitro* filter-binding assay but had increased specificity against the FLAG and hDlg control binding reactions (Figure **3F,G**).

### Confirmation in HPV-dependent cell culture models

**Figure 5:**
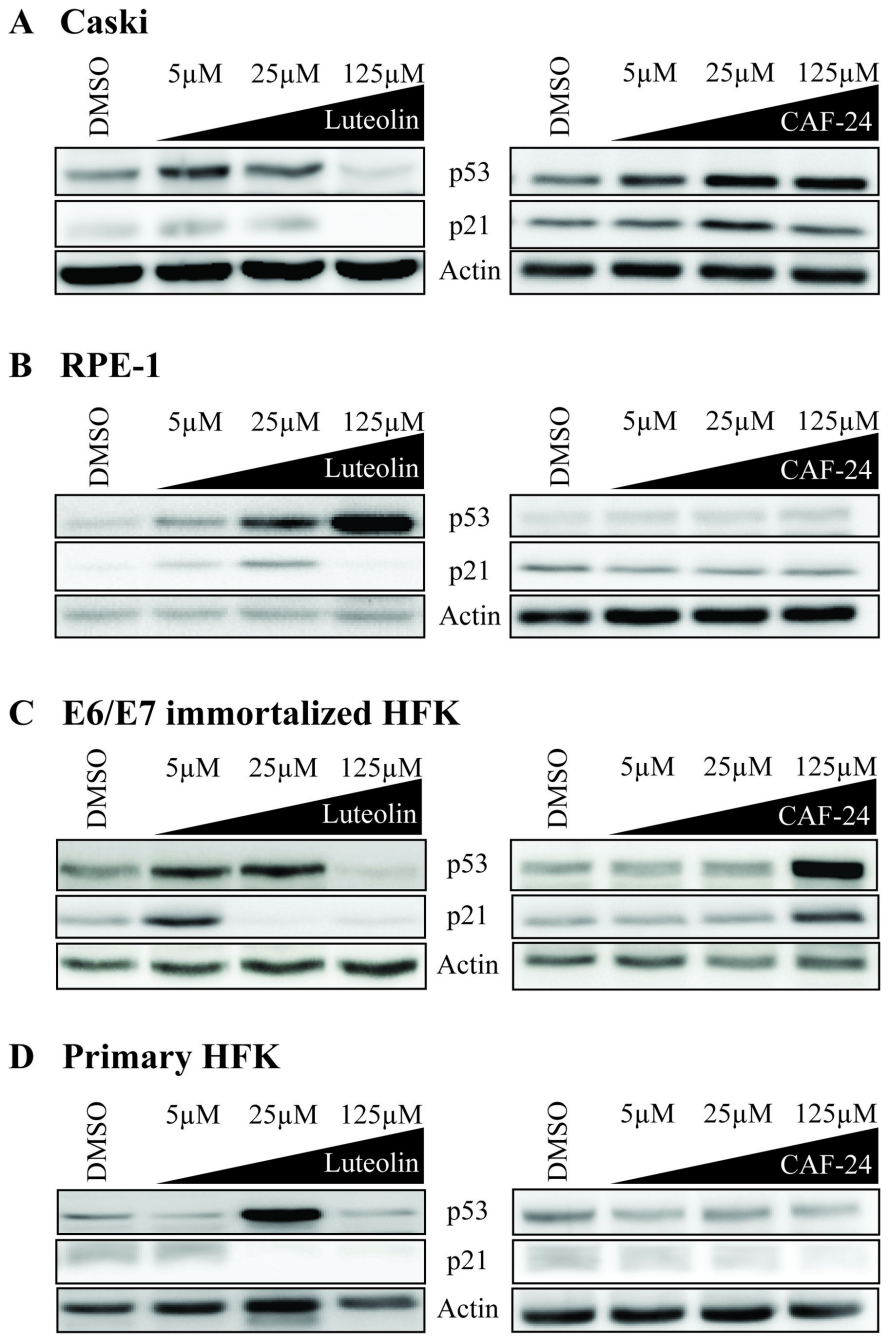
Compound activity in HPV positive cell lines. Compounds were tested for the ability to induce p53 and p21^Cip1/Waf1^ expression in Caski (**A**), RPE-1 (**B**) E6/E7 immortalized human foreskin keratinocytes (HFKs) (**C**), and primary HFKs (**D**). Actin was used as a loading control. Cells were incubated with compound for 40 hours.

A critical validation step was to confirm that the *in vitro* inhibitors demonstrate activity in cell-based assays. Two cell lines, one HPV positive and one HPV negative, were cultured in the presence of increasing concentrations of luteolin or CAF-24. Caski cells, which are derived from an HPV-16 infected cervical carcinoma and express HPV-16 E6 and E7, were used to determine whether these compounds interfere with HPV-16 E6 function. Inhibition of E6 should block its ability to promote the degradation of p53 thereby increasing the levels of p53. Treatment of Caski cells with each of these compounds led to increased p53 levels and a concomitant induction of the p53 responsive, cell cycle regulatory protein p21^Cip1/Waf1^ (Figure **5A**). These compounds were also assayed in telomerase immortalized human retinal epithelial cells (RPE-1). RPE-1 cells do not contain HPV DNA or express the E6 protein. If the elevation in p53 is dependent on the inhibition of E6 by these compounds, p53 should not increase in the RPE-1 cells. With luteolin, we did observe an increase in p53 protein levels, but only at concentrations higher than was required to increase p53 and p21 levels in the Caski cells (Figure **5B**). This is not unexpected as flavonoids such as luteolin have been identified as potential anticancer and anti-proliferative agents in a wide range of cancer cell lines [44–46]. Unlike luteolin, CAF-24 did not induce increased levels of p53 or p21 proteins in the HPV negative RPE-1 cell line (Figure **5B**).

These data were corroborated using human foreskin keratinocytes (HFK). Each compound was used to treat primary HFKs or HFKs immortalized through the over-expression of HPV16 E6 and E7 proteins [26]. In contrast to the E6/E7 immortalized HFKs, primary HFKs should not be dependent on E6 expression and should not demonstrate an increase in p53 in response to treatment with E6 inhibitors. As expected, both compounds increased p53 and p21 protein levels in the E6/E7 immortalized HFKs (Figure **4C**), although CAF-24 was less potent. As seen with the RPE-1 cells, luteolin promoted an E6 independent increase in p53 and p21 in the primary keratinocytes (Figure **5D**), whereas CAF-24 had little effect on p53 and p21 levels (Figure **5D**).

**Figure 6:**
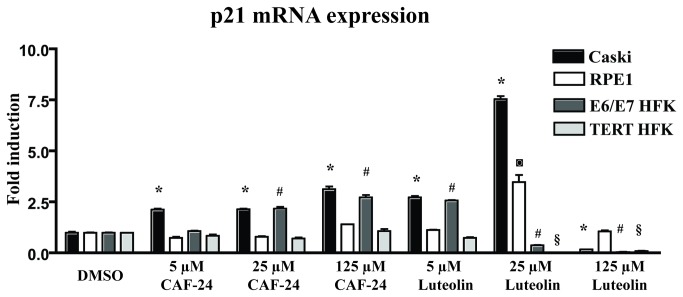
Induction of p21^Cip1/Waf1^ mRNA. Compounds were tested for the ability to induce p21^Cip1/Waf1^ expression at the mRNA level. Relative increases in transcripts were determined by qRT-PCR. Caski (black), RPE-1 (white), E6/E7 immortalized HFKs (gray), and hTERT immortalized HFKs (light gray) cells were treated at 3 increasing concentrations of either CAF-24 or luteolin for 24 hours. Fold induction was calculated in relation to treatment with DMSO and normalized to GAPDH. Data were analyzed using one-way ANOVA with post-hoc analysis (Dunnetts; S.E.M.; n=2). Statistical significance (p<0.01) is indicated as * in comparison to Caski control (DMSO), # E6/E7 HFK control (DMSO), § HFK control (DMSO), and ◙ for RPE-1 control (DMSO).

It is possible that the increased p53 and p21 levels resulted from non-specific inhibition of protein degradation. However, if the increase in p21 was dependent upon p53 promoter activation, it should be possible to detect changes in the amounts of p21 mRNA detected in treated cells. To confirm that the increase in p21 protein level resulted from p53 activation, the levels of p21 mRNA were analyzed using qRT-PCR. The increase in p21 mRNA observed with luteolin and CAF-24 treatment mirrored the increase in p21 protein (Figure **6**), supporting the hypothesis that the increase in p21 protein resulted from an increase in the amount of p53. 

**Figure 7:**
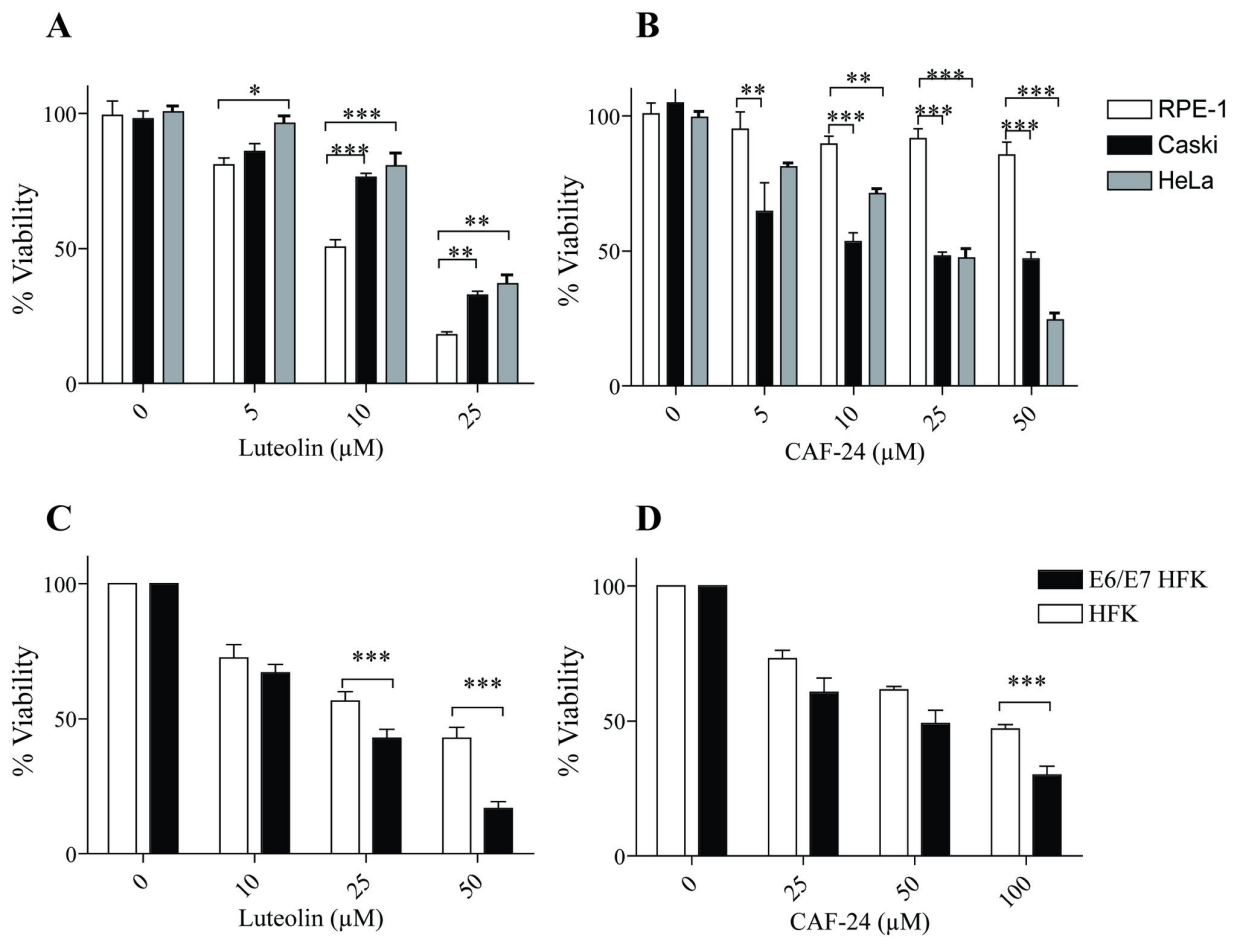
Effects of E6 inhibitors on cell viability. Compounds were tested for the ability to change cell viability. Caski (black), HeLa (gray), and RPE-1 (white) cells were treated with either luteolin (**A**) or CAF-24 (**B**) for 72 hours. HFKs (white) or E6/E7 immortalized HFKs (black) were treated with luteolin (**C**) or CAF-24 (**D**) for 48 hours. Cell density was determined using Celltiter 96 AQueous one solution assay. Percent viability was determined for each sample relative to the DMSO treated control samples. Viability in each HPV positive cell line was compared to an analogous HPV negative cell line to determine if the compounds selectively inhibited growth in HPV positive cell lines. Caski and HeLa were compared to RPE-1 (**A**,**B**). E6/E7 HKFs were compared to primary HFKs (**C**,**D**). Significance was determined by 2-way ANOVA (n=3; S.E.M.; p <0.5 =*, p<0.1= **, and p<0.05***).

HPV-18 positive HeLa cells and two additional control cell lines, C33a, a cell line derived from an HPV-negative human cervical cancer isolate, and HaCat, a spontaneously immortalized human keratinocyte cell line, were used to assess compounds for their impact on cell growth and viability. All cells were incubated for 48 to 96 hours with increasing doses of compound. Cell viability was measured by MTS based assay and % viability quantified relative to cells treated with DMSO. Luteolin was a potent inhibitor of Caski, E6/E7 HFKs, and HeLa growth, but they also decreased viability in the control cell lines (Figure **7A,C** and Figure **S1**). This is in agreement with the lack of specificity for HPV-dependent cells observed in the p53 western results. Specificity for HPV positive cells was observed with CAF-24 treatment. CAF-24 selectively inhibited growth of Caski, E6/E7 HFKs, and HeLa cells with little effect on the viability of the HPV negative cell lines C33a, HaCat, and RPE1 (Figure **7B, D** and Figure **S1**). CAF-24 did, to a lesser extent, inhibit growth of the primary HFKs, but the difference in viability between primary HFKs than E6/E7 HFKs was statistically significant (Figure **7D** **,** p< 0.05).

### Senescence, cell morphology, and apoptosis

The increased p53 and p21 levels suggested that the decrease in cell viability might be due to p53-induced senescence or apoptosis. Caski, HeLa, RPE-1, C33a, and HaCat cells were treated with luteolin or CAF-24 for 72 hours, fixed, and stained for senescence associated beta galactosidase (SA-β-gal) activity. Caski cells had low-level basal β-gal staining in vehicle treated samples that increased more than two-fold following treatment with CAF-24 (Figure **S2A**). This was specific to Caski cells and was not observed in RPE-1, HeLa cells, or the HPV negative control cell lines C33a and HaCat (Figures **S2A,B**). Luteolin treatment caused a dramatic decrease in the number of cells per well. As observed in the viability assay, this occurred in both the HPV positive and negative cell lines. We observed no significant increase in β-gal staining in any of the cell lines in the presence of luteolin. While CAF-24 decreased Caski and HeLa cell numbers, no change in cell morphology was observed in any cell type. Luteolin caused severe morphological changes in most cell lines including vacuole formulation in HeLa, Caski, C33a, and HaCat cells, and the formation and extension of processes in RPE-1 culture (Figure **S2A** **;** luteolin @ 50µM). These cell lines were also assayed for apoptosis. Each was cultured in 96-well dishes in the presence of CAF-24 or luteolin from 4 – 48 hours and tested for activity of caspases 3 and 7. We did not observe increased caspase activity with either compound at any time point (data not shown). 

### *In vitro* p53 degradation

**Figure 8:**
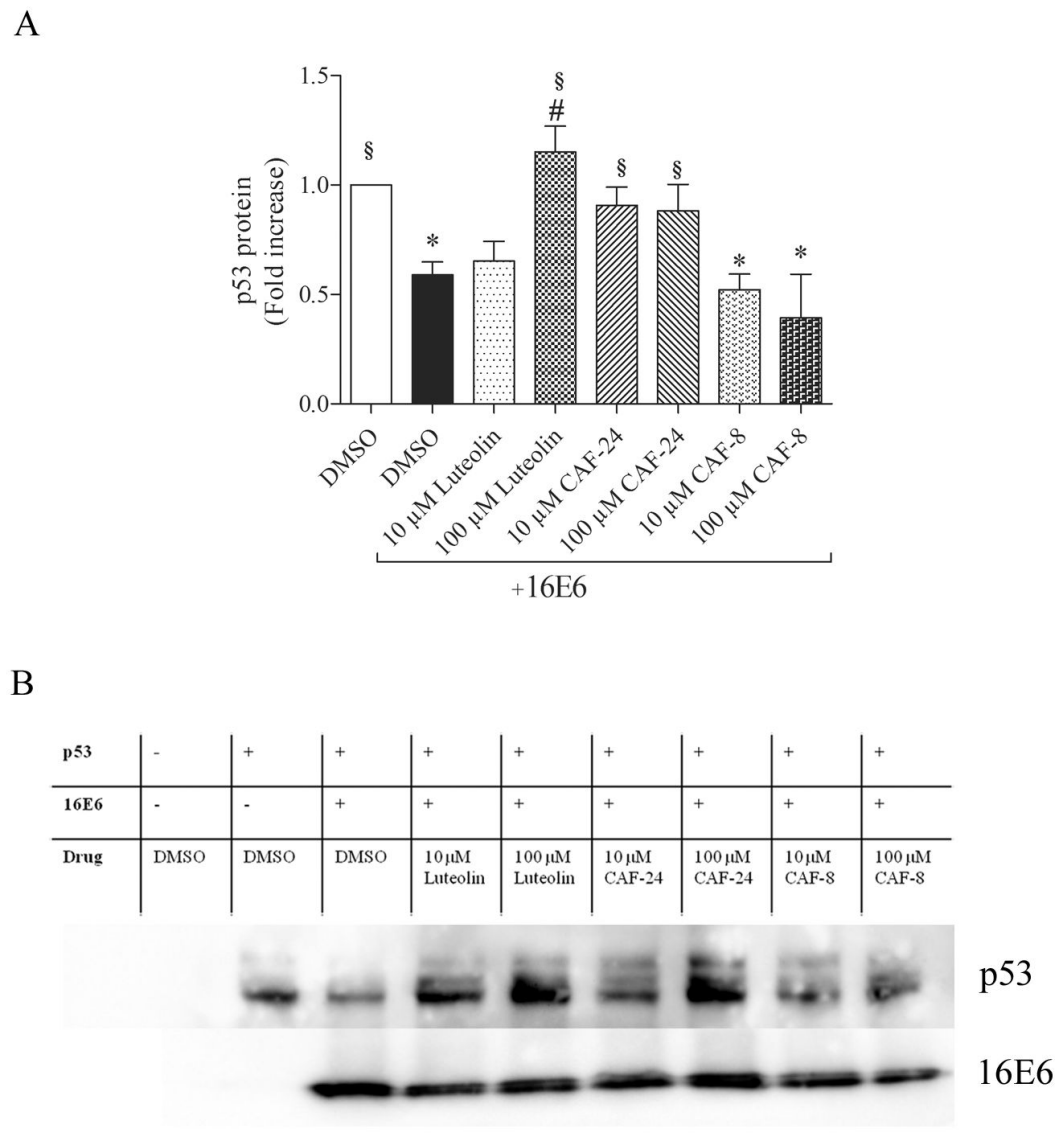
Activity of flavonoid derivates on HPV-16 E6 mediated p53 degradation *in vitro*. Luteolin, CAF-24 and CAF-8 were tested for *in* *vitro* activity to alter 16E6 mediated p53 degradation. (**A**) p53 signal intensities are expressed as fold increase over p53 control (DMSO). Data were analyzed using one-way ANOVA with post-hoc analysis (Dunnetts; S.E.M.; n=5). Statistical significance (p<0.05) is indicated as * in comparison to p53 control (DMSO), # to p53 and 16 E6 (DMSO) and § to p53 and 16E6 (CAF-8; 10, or 100 µM). (**B**) Representative Immunoblot of p53 and 16E6.

To confirm that these compounds inhibit the E6-dependent degradation of p53 directly, luteolin, CAF-24 and CAF-8 were tested by *in vitro* HPV-16 E6 mediated p53 degradation assay. *In vitro* expressed p53 was incubated in rabbit reticulocyte lysate in the presence or absence of *in vitro* expressed HPV16 E6. p53 levels were reduced in reaction containing E6 proteins (Figure **8**). This decrease in p53 was not observed when E6 proteins were pre-incubated with 100 µM luteolin. CAF-24 addition at both 10 and 100 µM also stabilized p53. Although this effect was not as pronounced as the effect of luteolin, it was consistent across both concentrations. CAF-8, the tetramethyl ester of luteolin, did not alter E6 mediated p53 degradation, which is consistent with the observed absence of activity in the E6/E6AP-BAP assay (Figure **3** and Table **S2**). 

### Direct binding between luteolin and E6

These compounds were identified on the basis of their similarity to the pharmacophore for the charged leucine helical E6 binding motif. To confirm a direct interaction between E6 and the compounds, a surface plasmon resonance binding assay was used. These experiments were limited due to the poor solubility of both luteolin and CAF-24 in simple aqueous solution [47]. Luteolin was soluble in the appropriate buffers at concentrations of less than 20 µM. 

**Figure 9:**
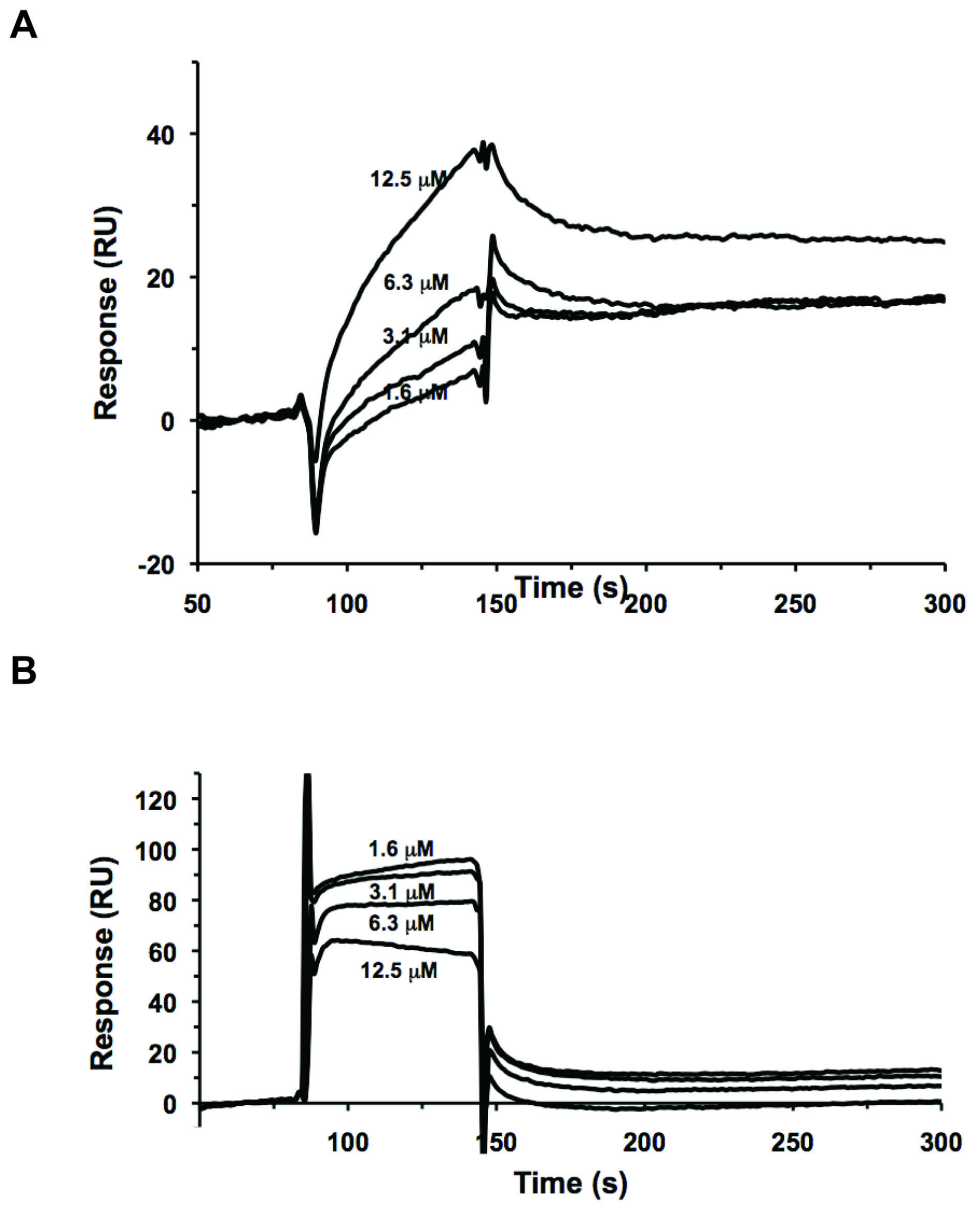
Direct binding between luteolin and E6. (**A**) In this SPR experiment, E6 was immobilized while luteolin was flowed through the cell. (**B**) SPR competition experiments using immobilized E6 ligand (E6apc2 peptide) competing for binding E6 protein (20 μM, pH 8) with various concentrations of luteolin.

For this study, E6 was fused to a GB1-fusion tag (GBF) and immobilized while luteolin was flowed through the cell at concentrations ranging from 1.6-12.5 µM. A reference cell with immobilized GBF was performed simultaneously for background subtraction. As luteolin concentration increased, the corresponding binding response increased (Figure **9A**), suggesting luteolin directly binds to E6 protein. While direct binding was observed, the data could not be properly fit to determine binding affinity. 

Luteolin was also able to compete for direct binding with an E6-binding peptide, E6apc2. The E6apc2 peptide was derived from the third zinc finger of the Sp1 protein and incorporates 10 amino acids from the charged helical domain of E6AP (**LQELL**GEER) at its C-terminus [17]. This peptide disrupts the interaction between E6 and E6AP with an IC_50_ of 19 µM [17]. In this experiment, E6apc2 was immobilized while E6apc1 control (E6-non-binder) was immobilized in a reference cell. GBF-E6 pre-incubated with luteolin in a dose range from 1.6 - 12.5 µM and was flowed as analyte solution. As the concentration of luteolin increased, a lower response for E6 binding to E6apc2 was observed (Figure **9B**) suggesting that luteolin directly competed with the peptide for binding to GBF-E6.

### Effect of flavonoids derivatives on MBP-E6 protein melting curve

**Figure 10:**
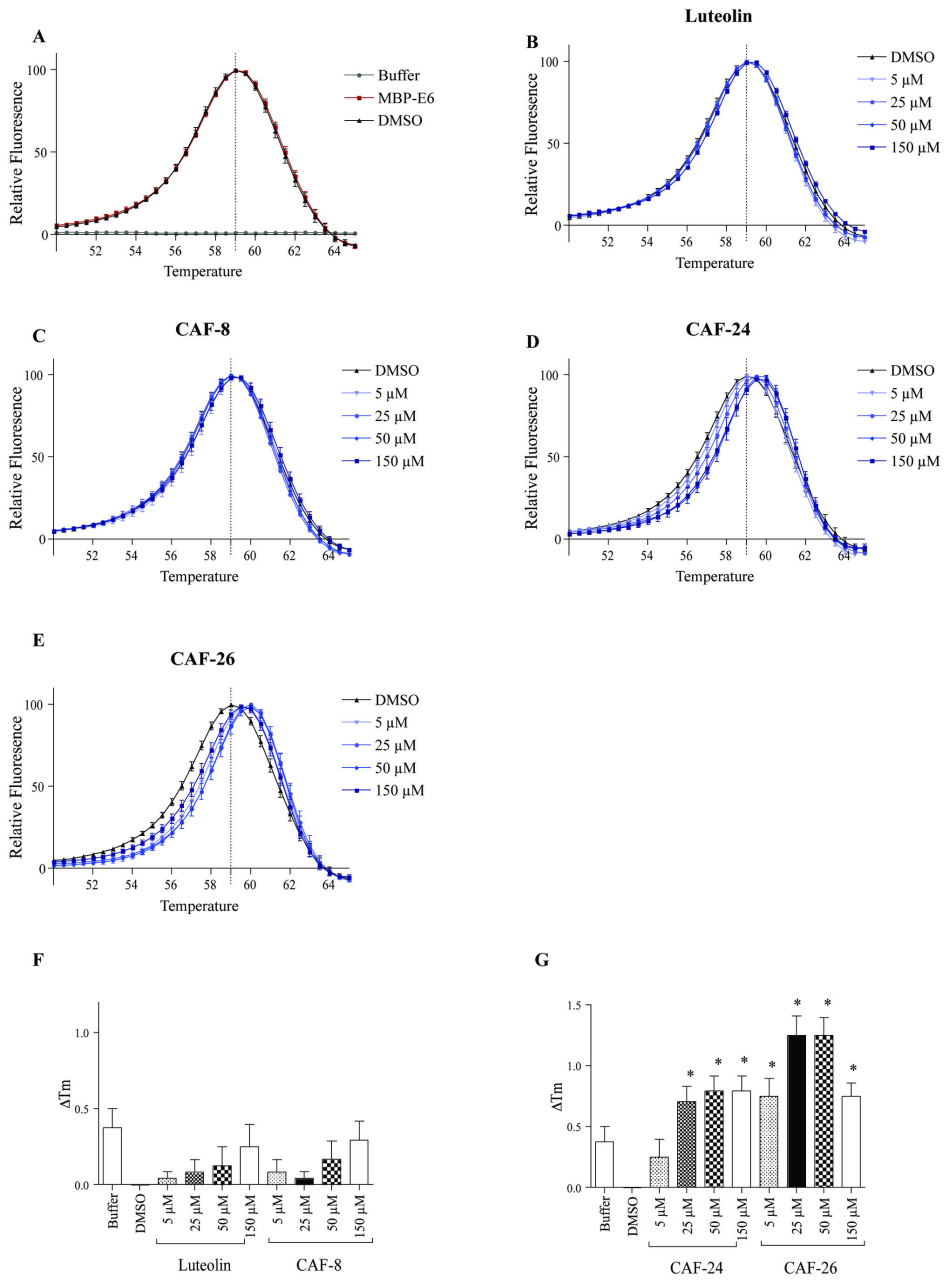
Effect of flavonoid derivates on thermal stability of MBP-E6 proteins. Melt curves of MBP-E6 proteins were measured using Sypro Orange dye. MBP-E6 proteins were equilibrated with luteolin, (5-150 µM), CAF-8 (5-150 µM), CAF-24 (5-150 µM),CAF-26 (5-150 µM) or DMSO (1.8% v/v) followed by addition of Sypro Orange and fluorescence measurement in a real-time PCR cycler. (**A**) Thermal fluorescence profile of MBP-E6 in assay buffer (square) or DMSO (triangle) along with assay buffer (circle). Dose effect of luteolin (**B**), CAF-8 (**C**), CAF-24 (**D**), and CAF-26 (**E**) on MBP-E6 protein melt curves. Vertical dotted line corresponds to MBP-E6 (DMSO) melting temperature. (**F**,**G**) Change of protein melting temperatures (∆Tm) of MBP-E6 by assay buffer, luteolin, CAF-8, CAF-24 and CAF-26 over DMSO. Data were analyzed using one-way ANOVA with post-hoc analysis (Dunnetts; S.E.M.; n=4). P<0.05 was considered as statistical significant and * indicates statistical significance compared to DMSO treatment.

A thermal shift assay was used to assess compound binding to HPV E6. In this reaction, protein denaturation is measured by an increase in fluorescence from Sypro Orange as it binds to the newly exposed hydrophobic surfaces on the protein [48]. A protein stability curve is created as a measure of the rate of protein unfolding. The relative fluorescence is plotted against temperature and the melting temperature (Tm) is determined by calculating the temperature of peak fluorescence. Ligand binding can stabilize the protein and increase the Tm. The thermal profile of MBP-E6 proteins was investigated to determine if the flavonoid derivatives could stabilize MBP-E6 protein structure during heat-induced unfolding. The thermal profile of MBP-E6 in assay buffer compared to DMSO (1.8% v/v) revealed that DMSO shifted the Tm of MBP-E6 from 59.38 ± 0.13°C to 59.0 ± 0.0°C (Figure **10A**), which failed to reach statistical significance. CAF-8, which did not inhibit the E6-E6AP interaction *in vitro* (Figure **8**), was unable to significantly alter MBP-E6 melting temperature at the tested concentrations (Figure **10C**). Both CAF-24 and CAF-26 were able to shift MBP-E6 Tm nearly 1°C at 25, 50 and 150 µM (Figure **10D,E**), indicating that these compounds bind to and stabilize MBP-E6 protein structure. Addition of luteolin had no effect on the Tm of MBP-E6 (Figure **10B**). The change in Tm (∆Tm) was plotted for each compound and compared to DMSO (Figure **10F,G**). Only CAF-24 and CAF-26 had a significant effect on the Tm for MBP E6.

### Docking of flavones into the E6AP binding site on the three dimensional structure of HPV16-E6

**Figure 11:**
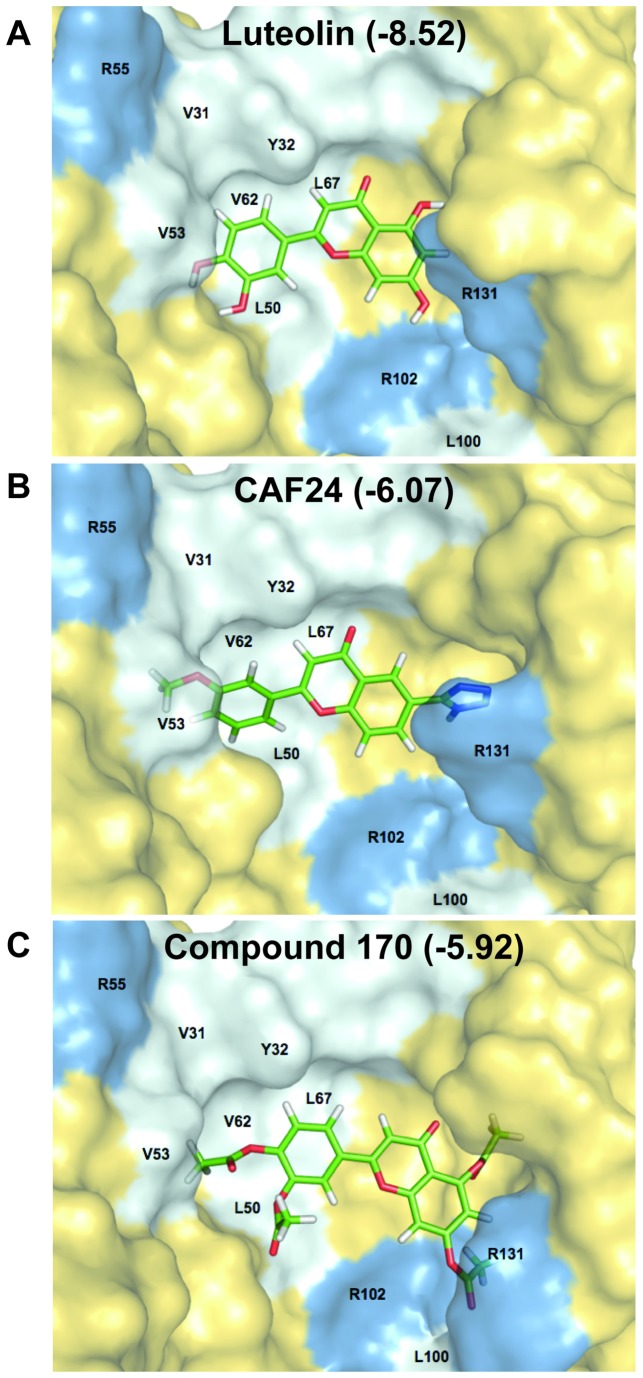
Induced-fit docking of flavone compounds with E6. Starting from the crystal structure of HPV E6 bound to the LXXLL peptide of E6AP (PDB ID: 4GIZ), luteolin (**A**), CAF-24 (**B**) and the acetylated analog of luteolin; Compound 170 (C) were flexibly docked into E6 using the induced-fit docking model in Schrödinger Maestro, while allowing protein flexibility within a 10Å radius of each ligand. Amongst the three flavone compounds, luteolin and CAF-24 docked in similar conformations and appear to interact with E6 in a comparable fashion. Compound 170, however, was unable to bind in the same way as the other two compounds, likely due to steric hindrance. The respective GLIDE score (kcal/mol) of each compound is shown for each docked structure.

Luteolin, CAF-24, and the acetylated luteolin pro-drug were docked as ligands onto the E6 structure (PDB ID: 4GIZ) [38] using an induced-fit docking model in Schrödinger Maestro [49,50] (Figure **11**). Each compound was docked flexibly in the center of the interaction space using a predefined grid in GLIDE [51,52]. In preparation of docking, the crystal structure of HPV E6 bound to the five amino acid residues of E6AP (maltose-binding periplasmic protein was removed) was minimized using the OPLS2005 force field. Ligands were prepared using LigPrep and also minimized with the OPLS2005 force field. For the induced-fit model, protein amino acid conformation sampling was performed within 20Å of each ligand, and the ligands were re-docked using the GLIDE XP algorithm. 

We compared the GLIDE scores (kcal/mol) for the selected compounds with their inhibitory activity for HPV-16 E6 in the *in vitro* binding assay. The lower the score, the higher the predicted affinity between E6 and the compound. In this analysis, luteolin has a GLIDE score of -8.52 kcal/mol and appears to bind at the binding interface for the charged leucine helix of E6AP. As would be predicted from our binding assay analysis (Table **S1**), the GLIDE score for the luteolin pro-drug, compound 170, was higher than that of luteolin. Despite having an affinity for E6 that is similar to luteolin in the binding assay, CAF-24 had a GLIDE score of -6.08. This would predict a less active compound with a lower affinity for E6, but a possible reason for this is discussed below. Each of the compounds docks to the hydrophobic pocket in HPV-E6 that corresponds to the residues L70, V62, and L67. 

## Discussion

An accurate structure of the LxxLL binding surface on HPV-16 E6 was not available when we began these investigations. Therefore, we chose to focus our efforts on the structure of the LxxLL helical motif [15,16]. We previously reported a pharmacophore derived from the three-dimensional structure of the conserved LxxLL domain from the E6AP and E6BP proteins could be used to identify peptidomimetic compounds that disrupt the interaction between E6 and E6AP and block the E6-dependent degradation of p53 [18]. This work served as proof of principle but the identified compounds lacked potency and were not tractable to chemical modification. We report here the results of a second *in silico* screen using the NCI collections using a more constrained four-point pharmacophore. Hits were confirmed in a multiwell plate based *in vitro* E6-E6AP binding assay and selected compounds were entered into an iterative chemistry program to identify more active and potent analogs using the E6-E6AP binding assay. 

An early model of the full-length structure of E6 was derived from the C-terminal half of the molecule [53]. This model was based on the assumption that the two halves of E6, which each contain a zinc binding motif, would adopt similar folds and form a pseudodimer along hydrophobic patches in each half of the molecule. However, this model was not able to fully explain many HPV-16 E6 mutants that disrupted the interaction between E6 and E6AP and the degradation of p53 [43]. A more recent NMR analysis of a dimerization mutant of the full length HPV-16 E6 protein has been determined [47]. The spectra matched well with that of the individual N- and C- terminal halves of the molecule, however conformations of the N-termini of each half do not change in the full-length context and is inconsistent with a previous pseudo-dimeric model [53]. The authors proposed that E6 captures LxxLL charged helix domains through its interaction with the exposed hydrophobic patch on the surface of the molecule. This was consistent with the structure of BPV1 in complex with the LxxLL domain of paxillin [38,54]. 

**Figure 12:**
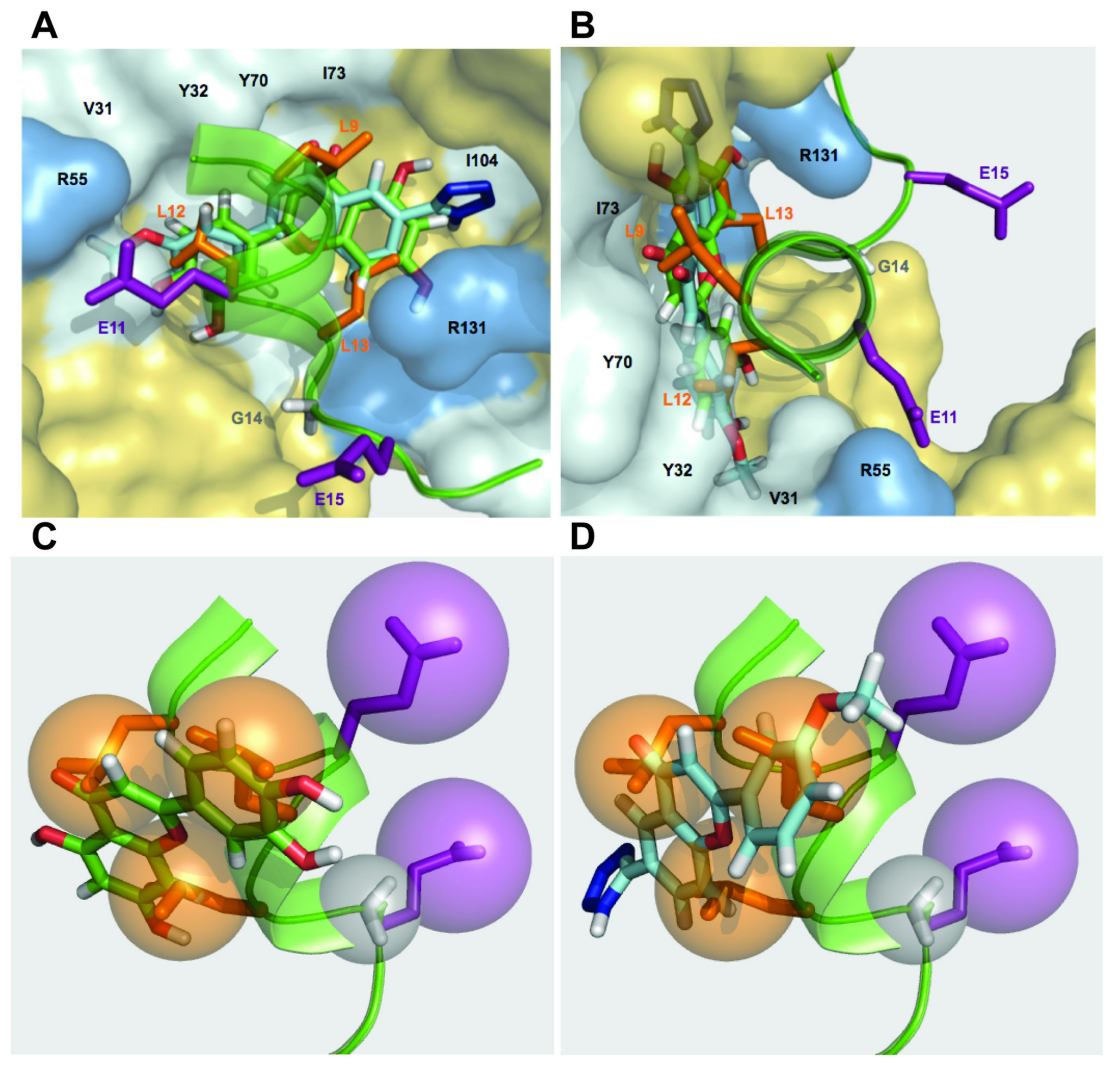
Flavone compound docking and pharmacophore comparison. (**A**) Illustration of E6AP (green helix) binding to HPV16-E6 (PDB ID: 4GIZ). Important residues in E6 are highlighted. Hydrophobic residues are gray, and selected arginine residues are blue. The residues included in our pharmacophore (L9, E11, L12, L13, G14, and E15) are labeled. Leucines are orange, glutamic acids are purple, and glycine is gray. CAF-24 and luteolin are overlaid on this model. (**B**) Using the docking models and the positioning of the E6AP helix, luteolin (**C**) and CAF-24 (**D**) are docked on to the six-point E6-binding pharmacophore. Each of the compounds makes contact with 4 of the 6 interaction spheres defined in this model, E11, L12, L13, and G14.

The three-dimensional crystal structure of HPV-16 E6 in complex with E6AP has now been determined [38]. The authors predict key molecular contacts between E6 and the α-helical motif of E6AP. Residues that appear to be involved in this interaction include R10, V31, Y32, L50, R55, V62, L67, Y70, I73, R102, and R131 (Figures **11** and **12**). The residues at positions 31,32, 50, 62, 67, 70, and 73 are part of the hydrophobic surface at the E6/E6AP binding interface. The arginine residues appear to interact with the outer, charged face of the α-helical motif of E6AP. We propose that compounds that bind to this surface in E6 can prevent its association with E6AP and will inhibit E6-mediated p53 degradation. Such E6 inhibitors could be developed as HPV antiviral therapies as well as potential treatments for HPV related cervical cancer. We have identified a series of flavonoids that can bind to E6, inhibit p53 degradation, and decrease viability of HPV positive cell lines. Interestingly, other naturally occurring flavonoids were identified as potential E6 inhibitors in an assay using caspase 8 binding to HPV-16 E6 [55]. 

Luteolin, CAF-24, and CAF-26 disrupted the interaction between E6 and E6AP in the *in vitro* binding assay with low micromolar IC_50_ values (Figure **3**). The tetramethyl analog of luteolin, CAF-8, was inactive in this assay. This appears to be due to loss of hydrogen bonding potential by masking of the hydroxyls in the A’ and B rings of the flavone scaffold. The inhibition of the interaction of E6 and E6AP correlated with a decrease in E6-dependent degradation of p53 *in vitro* (Figure **8**). Luteolin and CAF-24 prevented p53 degradation in this assay, while CAF-8 displayed no inhibitory activity. These observations translated well to studies in HPV-positive cell lines (Figures **5**-**7** and Figures **S1,S2**). CAF-24 increased p53 levels and decreased cell viability in HPV positive cell lines. Luteolin also increased p53 levels and decreased cell viability but lacked specificity and displayed off-target toxicity in HPV negative cells. 

In surface plasmon resonance assays, luteolin bound directly to E6 and displaced the E6AP helical motif from its complex with GBF-E6 (Figure **9**). Due to limited aqueous solubility CAF-24 and CAF-26 could not be screened in this assay. There was also a strong correlation between E6 inhibition *in vitro* and MBP-E6 stability in thermal shift assays. The novel flavones CAF-24 and CAF-26 increased the Tm of MBP-E6 by about 1°C (Figure **10**), while CAF-8 had no measurable effect. While luteolin was tested in this assay, it did not shift the Tm of MBP-E6. The disagreement between SPR and thermal shift data may be explained by the small size and potential lack of specificity of luteolin. The affinity of luteolin for the E6AP interaction surface and stabilizing effect on MBP-E6 folding may be too small to detect. It is also possible that the hydrogen bonding potential of luteolin may allow it to bind equally in native and unfolded states thus masking any shift in Tm. 

The compounds luteolin, 170, and CAF-24 were virtually docked onto the three-dimensional structure of E6 using the induced-fit docking model in Schrödinger Maestro [49,50]. This analysis predicted that the compounds bind in a hydrophobic pocket at the interface between E6 and E6AP (Figure **11**). It is interesting to note that in Figure **11C** the tetrazole group of CAF-24 extends into a pocket created between R131 and I104 in E6. This additional group may restrict CAF-24 from binding to off-target hydrophobic sites and explain the difference in specificity between luteolin and CAF-24. It is noted, however, that these compounds did not quantitatively rank with their respective experimental IC_50_ values when compared to one another, as can be seen with their respective GLIDE scores (Figure **11**). This could be explained by the observation that tetrazole-containing compounds would often adopt alternative orientations in the E6AP binding site on E6 when interrogated using GLIDE. Ionized tetrazole groups would orient towards L100, potentially indicating the presence of multiple possible modes of binding. This factor would not be represented in a compound’s IC_50_ score as it could still bind at the site of interaction and disrupt E6-E6AP binding, regardless of its affinity for a particular orientation at that site. Although tetrazole functional groups are likely to be ionized at physiological pH, this does not necessarily preclude their protonation and binding in the orientation as depicted in Figure **11C**. 

By overlaying the docking models for luteolin and CAF-24 with the resolved structure for HPV16-E6 bound to the E6AP α-helical motif, we can visualize how the compounds fulfill the molecular contacts defined by the pharmacophore (Figures **2** and **12**). Luteolin and CAF-24 dock in the hydrophobic pocket and are oriented so that the A and A’ rings fill the same space as the leucines on positions 9, 12, and 13 of the E6AP helix (Figure **12A**). Based on the docking model neither of the compounds mimics the charged groups represented by the glutamic acid residues at positions 11 and 15. This observation is visualized down the axis of the helix (Figure **12B**). 

The resolved structure predicts that the glutamic acid at position 16 of E6AP interacts directly with the arginine at residue 10 in E6 [38]. In this structure, the E15 residue of E6AP projects out and away from the binding surface on E6AP and does not appear to be involved in binding. The glycine at position 14 allows the E6AP helix to pack between the arginines at positions 131 and 10 in E6. The flexibility at this position may also promote the interaction between the E16 of E6AP and R10 in E6. Neither compound extends into the exclusion sphere for G14. 

Based on this docking, our compounds match 4 of the six points in our pharmacophore at L9, L12, L13, and G14, thus reproducing 4 of the 6 contacts points in the LxxLLG core of the helical ‘charged leucine’ motif. If this model is correct, it is possible that the flavone core could be used as an anchor and reactive groups could be added to promote interaction with the arginine residues at positions 10 and 131. Not only would such compounds have increased affinity and specificity for E6, but the hydrophilic nature of such groups could also act to repel the hydrophobic surface of the charged leucine helix of E6AP and increase the potency of these compounds.

CAF-24 has a tetrazole in position 6. This substituent does not disrupt the binding but rather seems to increase specificity. The tetrazole group can act as a bioisostere of carboxylic acid [56] and may serve for additional contacts on E6; however, it is possible that the addition of the tetrazole increases the water solubility of the ligand, thus resulting in favorable changes in solvation/desolvation energies. In the docking model for CAF-24 shown in this study, the tetrazole group fits into a pocket between I104 and R131 (Figure **11C**). The size of this group may sterically hinder binding to proteins other than E6 and could be responsible for its increased specificity. Other novel flavone analogs have bulky substituents at either position 6 or 7 and maintain inhibitory activity (Figure **4**). We are continuing to explore the benefit of including such groups while attempting to increase the potency, activity and specificity of these compounds. 

The minor structural differences within this compound series accounts for dramatic changes in specificity. While the natural compounds, including luteolin, may prove valuable as chemical probes for studying the role of HPV E6 in cell models, the off-target effects in HPV-negative cells lines rules these out as candidates for therapeutic intervention in HPV infection and cervical cancer.

 There is no antiviral treatment for HPV infection. Current therapies for cervical dysplasia and cancer involve destruction or removal of the infected tissue by cytotoxic agents or surgery. While therapeutic HPV vaccines have been considered, efficacy in the anecdotal trials has been modest at best. Prophylactic HPV vaccines are very effective, nonetheless, there are major socioeconomic challenges to worldwide implementation, acceptance, and use of an expensive vaccine program. Moreover, the prophylactic HPV vaccine offers no benefit for the millions of women and men already infected with HPV. Because of the long latency between existing HPV infection and malignancy, a measurable reduction in cervical cancer will not occur for at least 10 years. The therapeutic intent of this project takes advantage of the fact that progression from dysplasia to malignancy occurs over several years, providing a window of opportunity for an effective medical treatment.

These studies should lead to the identification new chemical probes of the conserved regions within the LxxLL/E6 interaction surface, thereby illustrating druggable targets on the high-risk E6 proteins, and drive our current E6 inhibitor optimization program.

## Materials and Methods

### Definition of the pharmacophore and *in silico* screening

The pharmacophore used in this study is the E6AP model [18] and defines the spatial orientation of reactive groups in the E6AP helix, specifically L9, E11, L12, L13, G14, and E15. These are represented by three hydrophilic points (L9, L12, and L13), two hydrogen-binding points (E11 and E15), and an exclusion sphere (G14) (Figure **2A**). Unlike the previous report, which queried all 6 points in the pharmacophore, in this study sets of 4 points in the pharmacophore were compared to generate reasonable numbers of hits. *In silico* screening was otherwise performed as described previously except that both NCI and Synthline databases were queried [18]. 

### Expression and purification of GST-E6 and BAP fusion proteins

#### GST-E6

Expression – A 10 mL overnight starter culture of GST-E6 expressing bacteria (BL21) was transferred to 1L TurboBroth and grown at 37°C until it reached an O.D. of ~0.5. Cultures were induced overnight at 28°C with 0.2 mM IPTG and 0.3 mM lactose. Cells were pelleted at 6000x g and lysed in 50 ml of lysis buffer. Lysis buffer contained 100 mM Tris pH 8.0, 100 mM NaCl, 0.1% NP-40, 5.0 mM DTT, and 1 x protease inhibitor cocktail (EDTA free, Sigma). Cells were sonicated 6 times; 10 sec. ON and 30 sec OFF; at an amplitude of 40%. Lysates were cleared at 15,000x g for 30 minutes and transferred to a new tube. Purification – 1 mL of 50% glutathione bead slurry (GE Healthcare Life Sciences, Glutathione Sepharose 4 Fast Flow) was added to the lysate and incubated overnight at 4°C with inversion. Beads were collected by centrifugation at 500xg for 5 minutes and kept on ice. Beads were washed 4 times for 10 minutes with inversion at 4°C in ice cold lysis buffer without NP-40. After final wash, transfer to 1.5 mL tube and store in lysis buffer without NP-40. GST-E6 proteins were kept at 4°C for no more than 1 week. 

#### BAP fusions

Expression – A 10 mL overnight starter cultures of BAP fusion expressing bacteria (BL21) were transferred to 1L TurboBroth and grown at 37°C for 3-4 hours. No induction was required. Cells were pelleted at 6000x g, lysed in 50 ml of lysis buffer, and sonicated as described before. Lysates were cleared at 15,000x g for 30 minutes and transferred to a new tube. Purification – 250 µL of anti-flag resin (Sigma A2220) was added to the lysate and incubated overnight at 4°C with inversion. Beads were collected by centrifugation at 500xg for 5 minutes and kept on ice. Beads were re-suspended in lysis buffer without NP-40 and transferred to an Econocolumn (BioRad). The beads were washed with 20 mL of ice cold lysis buffer without NP-40. E6AP-BAP was released from the column by 5 successive elutions with 250 µL of ice cold 0.2 M glycine pH 2.5 directly into 250 µL of neutralization buffer [50% glycerol, 200 mM NaCl, 200 mM Tris pH 7.5, 2x EDTA free protease inhibitor cocktail (Sigma)]. BAP fusion proteins can be maintained at 4°C for < 2 weeks.

### *In vitro* filter binding assay

Pre-Block - 96-well filter plates were prepared (0.2 µm BioTrace NT, Pall). 100µL of blocking buffer (100 mM Tris pH 8.0, 100 mM NaCl, 0.1% milk) was added per well. Plates were blocked for 1 hr at RT on an orbital shaker. Buffer was removed by vacuum filtration using a vacuum manifold. GST-E6 Bead addition – GST-E6 beads were prepared at 5µg/mL in GST-E6 bead buffer (100 mM Tris pH 8.0, 100 mM NaCl, 0.1% milk, 2.5 mM DTT). 100µL of GST-E6 bead mix was added per well and incubated for 1 hr at 4°C. Buffer was removed by vacuum filtration. Binding Reaction – E6AP or hDlg BAP fusions were diluted to 50 nM or 25 nM respectively in binding buffer (100 mM Tris pH 8.0, 100 mM NaCl, 1 mM DTT). 48 µL of each reagent was added per well. For drug assay, 2µL of compound was added to each well and mixed thoroughly. The final reaction volume was 50 µL with 4% DMSO per well, which is well below the acceptable limits for DMSO in our binding assay. Plates were incubated for 1-3 hours at RT on an orbital shaker. Buffer was removed by vacuum filtration. Plates were washed on the vacuum manifold, first with 200 µL buffer (100 mM Tris pH 8.0, 100 mM NaCl, 1 mM DTT), and twice more with 100µL lysis buffer without DTT. Plates were cleared and dried with on the vacuum manifold. Assay – Immunostar assay reagent was prepared and equilibrated to RT prior to addition. 60 µL of Immunostar-AP assay reagent was added, the BAP activity was allowed to develop for 10 minutes and the plates were read on a Wallace Luminometer. BAP activity was measured as relative light units (RLU) on a luminometer using the Immunostar-AP substrate (BioRad). 

### Cell culture

Caski (ATCC CRL-1550), HeLa (ATCC CCL-2), C33a (ATCC HTB-31), and HaCat [57] cells were incubated in DMEM with 10% fetal bovine serum (Atlas). RPE-1 (ATCC-CRL-4000) cells were cultured in DMEM/F-12 with 10% FBS. HFKs, E6/E7 immortalized HFKs [26], and hTERT immortalized HFKs were cultured in Keratinocyte-SFM media (Life technologies, 17005-042).

### p53/p21 analysis

20,000 cells per cm^2^ were plated in 6-well dishes. Cells were incubated for 24 hours at which time fresh media and compound were added. After 40 hours, cells were harvested, washed with cold PBS, and lysed in RIPA buffer with protease inhibitor. Each sample was separated on a 12% SDS-page gel, transferred to Immobilon-P membrane (Millipore) and blotted for the p53 (DO-1, Santa Cruz), p21 (c-19, Santa Cruz), and actin (A2066, Sigma). Protein quantification was performed with the Fujifilm LAS-4000 Multifunctional Imaging System. The signal intensity was measured for each band on an immunoblot, normalized to the loading control, and the fold increase was determined in relation to the appropriate DMSO treated control. RNA was isolated from these cells using Trizol Reagent (Invitrogen 15596-026). cDNA was generated using the Improm-II Reverse Transcription System (Promega A3801). qPCR was performed as described in the protocol for SsoFast EvaGreen supermix (BioRad 172-5023) using an BioRad CFX96 real-time PCR machine. Melting curves for each reaction were obtained. Each sample was assayed in triplicate and every plate contained a 5-point cDNA dilution course to calculate amplification efficiency for each primer pair. The Pfaffl method was used to determine the change in transcript levels relative to the DMSO and normalized to GAPDH. 

### p53 *in vitro* degradation assay

p53 and E6 were *in vitro* translated using TNT® SP6 High-Yield Wheat Germ (Promega) according to manufacturer's instructions, as wheat germ does not contain E6AP proteins [10,29]. *In vitro* translated E6 proteins (1µL) were incubated with DMSO (2% v/v), Luteolin (10 µM, 100 µM), CAF-24 (10 µM, 100 µM) and CAF-8 (10 µM, 100 µM) in reaction buffer (25 mM Tris-HCl pH 7.5, 100 mM NaCl, 3 mM DTT, 2.5 mM ATP) at 30°C for 1 hr. Control reactions (no DNA, p53 alone) were incubated with 1 µL wheat germ extract and 2% DMSO in reaction buffer. *In vitro* translated p53 and rabbit reticulocyte lysate (RRL, Promega) were added to all reactions except the no DNA control reaction to which wheat germ extract was added instead of p53. Reactions were incubated for 3 hrs at 30°C and stopped by addition of 6X reducing SDS sample buffer and heating at 95°C for 5 min. Each reaction was loaded onto a 10% SDS-PAGE gel and transferred onto PVDF (0.45 µm). p53 and 16E6 were visualized using monoclonal p53 (Pab1801, 1:1000), monoclonal 16E6 (813, Arbor Vita Corporation, 1:4000) antibody and anti-mouse HRP-labeled secondary antibody (1:2000, Sigma-Aldrich). 

### Thermostability assay

MBP-16E6 DNA, a kind gift from G. Travé [58], was transformed into BL21DE(3) *E. coli*. Protein expression was performed by inoculation of a 1 mL starter culture in 100 mL of Turbo-Broth containing 0.2 % lactose, 0.05% glucose and 0.6% glycerol at 20°C for 24 hrs. Cells were lysed in 50 mM Tris HCl pH 6.8, 300 mM NaCl, 0.1 % (v/v) NP-40, 2 mM DTT and 1x protease inhibitor cocktail (EDTA-free, Roche) and sonicated as described before. Cell lysate was cleared by centrifugation at 15000xg, 4°C for 30 min and equilibrated with amylose resin beads (New England Biolabs, Inc.) by inversion for 3 hrs at 4°C. Bound beads were washed one time in lysis buffer and four times with wash buffer (50 mM Tris-HCl pH 6.8, 400 mM NaCl, 2 mM DTT, 1x protease inhibitor cocktail). Bound MBP-E6 proteins were eluted using a final concentration of 20 mM maltose in 5 bed volumes of wash buffer. All steps were performed at 4°C. Protein concentration was measured by BCA (BioRad). Thermostability of MBP-E6 proteins was analyzed at a final concentration of 1 µM in reaction buffer (50 mM TrisHCl pH 6.8, 400 mM NaCl, 2 mM DTT). MBP-E6 proteins were equilibrated with CAF-8 (5 µM-150 µM), CAF-24 (5 µM-150 µM), CAF-26 (5 µM-150 µM) or DMSO for 30 min in reaction buffer. Sypro Orange, a hydrophobic fluorescent dye, was added at the end of the incubation period at a final concentration of 5X. The final DMSO concentration (1.8% v/v) was kept constant across the concentration range. Fluorescence was measured using the FRET channel of CFX96 Real-Time PCR thermal cycler (BioRad) over a temperature range of 25°C to 95°C. Temperature increments were set to 0.5°C for 30 sec followed by plate reading. Melt curves were determined in triplicate and repeated four times. Melt curves represent the relative fluorescence of the 1st derivative of four experiments. Melting temperature (Tm) was calculated by CFX manager software. The average melting temperature of the triplicate was used to calculate the ∆Tm for each experiment. ∆Tm values are expressed as the change vs. DMSO. 

### Cell viability and caspase assays

For cell viability, 12,000 cells per cm^2^ were plated in 96-well dishes 24 hours prior to drug addition. Fresh media and compound were added. Plates were read after 72-96 hours. Relative cell viability was assayed as directed in the Celltiter 96 AQueous one solution assay (Promega). For caspase activity, plates were assayed after 4, 8, 12, 24, or 48 hours as described for the Caspase-Glo 3/7 assay (Promega). 

### SA-beta-gal assays

12,000 cells per cm^2^ were plated in 24-well dishes; after 24 hours, fresh media and compound were added. SA-beta gal staining was performed as described [59]. Images were collected using bright field microscopy. At least four fields (~100 cells per field) were read for each treatment, and experiments were repeated 3 times. The percent SA-beta-gal positive cells was calculated for each data point. 

### Surface plasmon resonance

SPR analysis was performed on a Biacore 3000 instrument. Immobilized GBFE6 [43] was diluted to 30μg/mL in 10 mM sodium acetate, pH 4.0 and 5.0 and immobilized onto the dextran matrix of a CM5 sensor chip (Biacore) using the amine coupling method. All binding experiments were performed at 25 °C in 50 mM phosphate (pH 6.5) containing 200 mM NaCl. Pulses of 10 mM glycine (pH 3.0) were used to regenerate the surfaces between injections. A 33-mer peptide, E6apc2 that binds E6, and a 29-mer peptide, E6apc1 that does not bind E6, were used as controls and are characterized previously [17]. SPR data were analyzed using BIAevaluation 4.1 software (Biacore). 

## Supporting Information

Figure S1 **Compounds were tested for the ability to alter cell viability.** Caski (black), HeLa (gray), RPE-1 (white), C33a (diagonal hash) and HaCat (crosshatch) cells were treated with either luteolin (**A**) or CAF-24 (**B**) for 72 hours. Cell density was determined using Celltiter 96 AQueous one solution assay. Percent viability was determined for each sample relative to the DMSO treated control samples.
(TIF)

Figure S2 **(**A**) For senescence associated βgalactosidase assays, HPV positive (Caski) and negative (RPE-1) cell lines were grown in the presence of CAF-24 or luteolin for 72 hours.** Cells were fixed and stained for SA-β-gal activity. (**B**) Senescence associated β-galactosidase assay results were quantified and are plotted as percent β-galactosidase positive cells. Additional data for β-galactosidase staining in C33a, HeLa, and HaCat are included.
(TIF)

Table S1 **Role of acetylation on activity and potency.**
(TIFF)

Table S2 **Role of methylation on activity and potency.**
(TIFF)

Table S3 **Role of the hydroxyls on activity and potency.**
(TIFF)
