# Supplementary figures and images for: Structure Based Identification and Characterization of Flavonoids That Disrupt Human Papillomavirus-16 E6 Function
Source: PLoS One. 2013 Dec 23;8(12):e84506. doi: 10.1371/journal.pone.0084506 (PMC3871595; doi:10.1371/journal.pone.0084506)

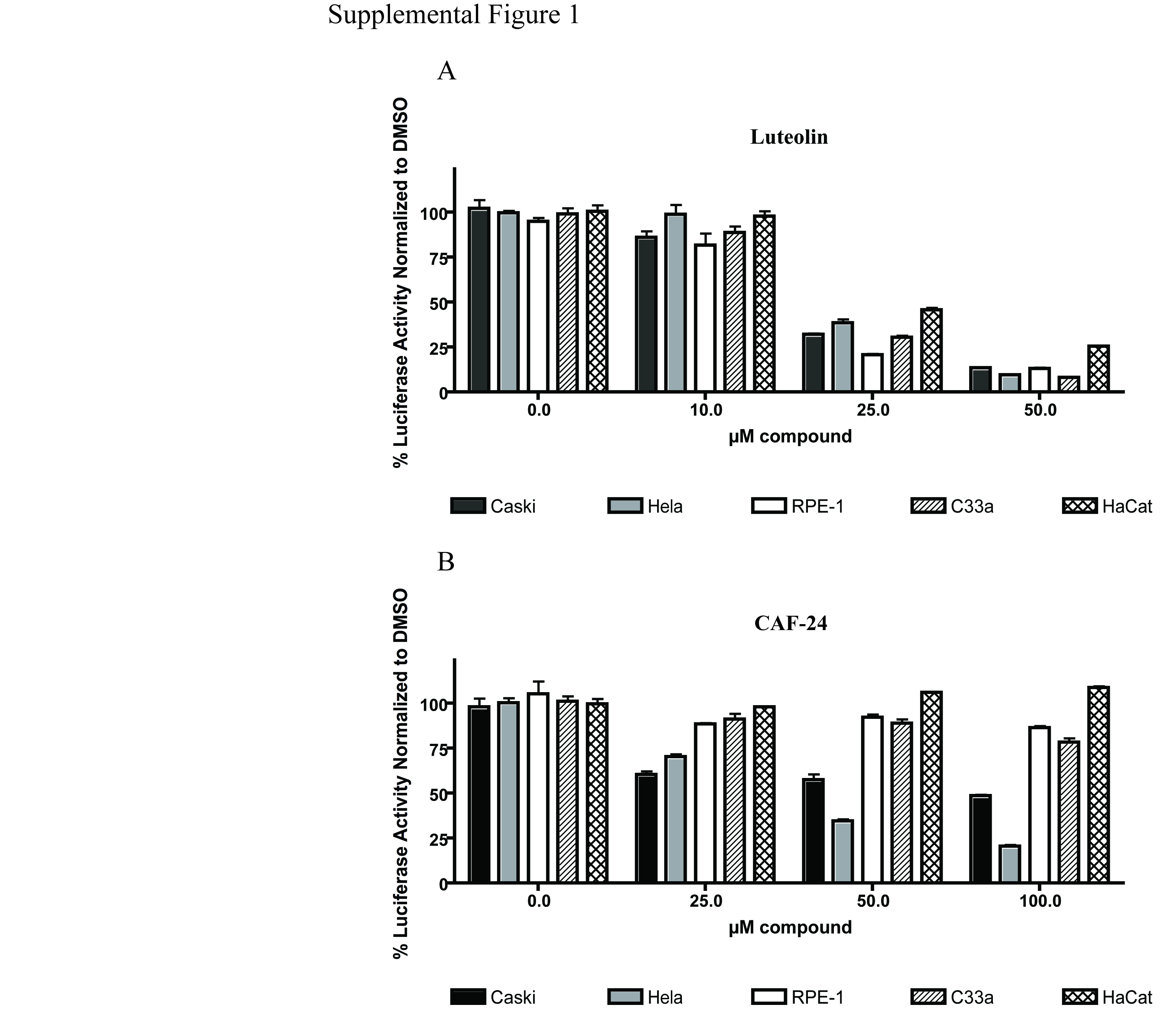

Supplement: Figure S1 — Compounds were tested for the ability to alter cell viability. Caski (black), HeLa (gray), RPE-1 (white), C33a (diagonal hash) and HaCat (crosshatch) cells were treated with either luteolin (A) or CAF-24 (B) for 72 hours. Cell density was determined using Celltiter 96 AQueous one solution assay. Percent viability was determined for each sample relative to the DMSO treated control samples. (TIF) [file pone.0084506.s001.tif]

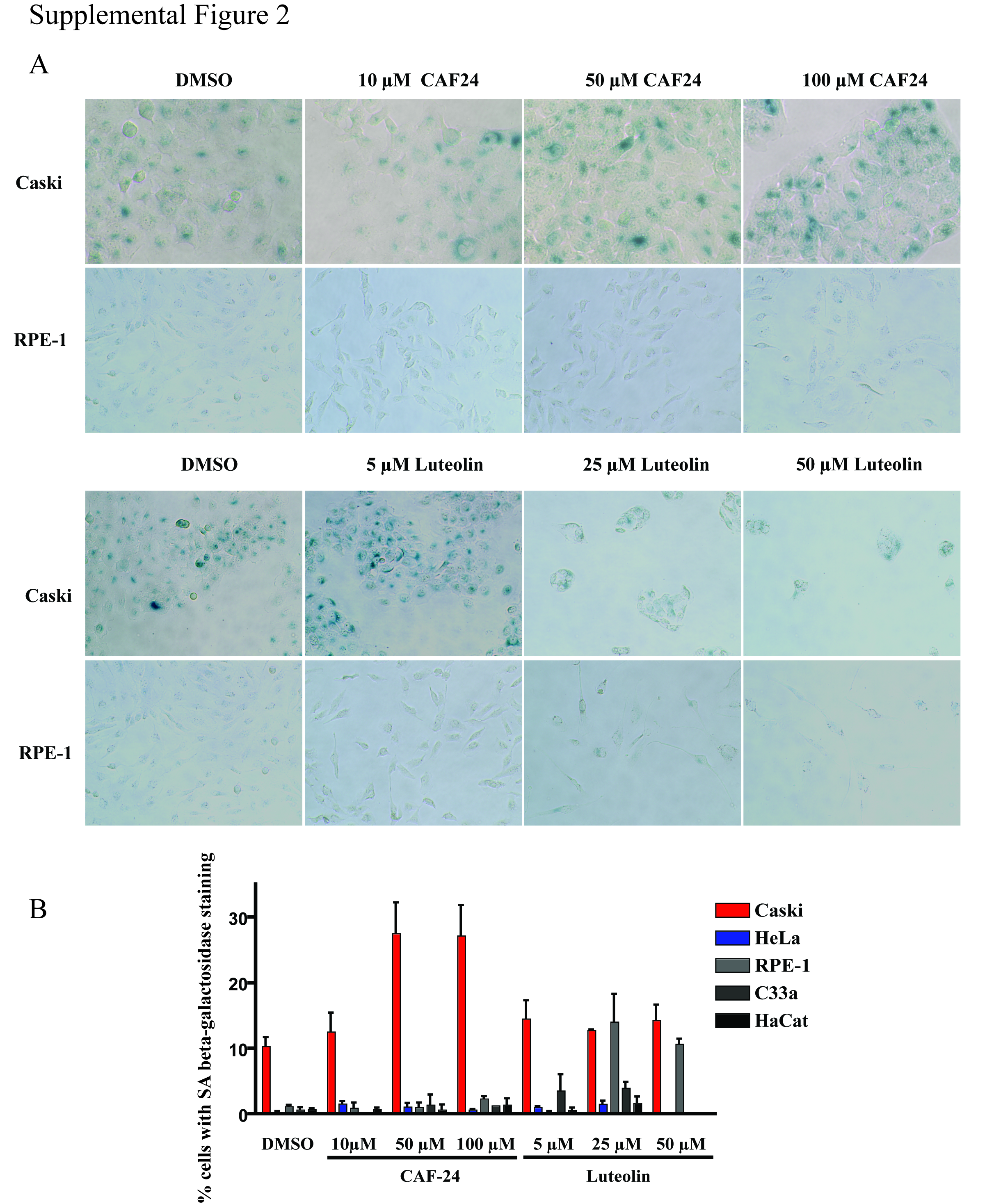

Supplement: Figure S2 — (A) For senescence associated βgalactosidase assays, HPV positive (Caski) and negative (RPE-1) cell lines were grown in the presence of CAF-24 or luteolin for 72 hours. Cells were fixed and stained for SA-β-gal activity. (B) Senescence associated β-galactosidase assay results were quantified and are plotted as percent β-galactosidase positive cells. Additional data for β-galactosidase staining in C33a, HeLa, and HaCat are included. (TIF) [file pone.0084506.s002.tif]

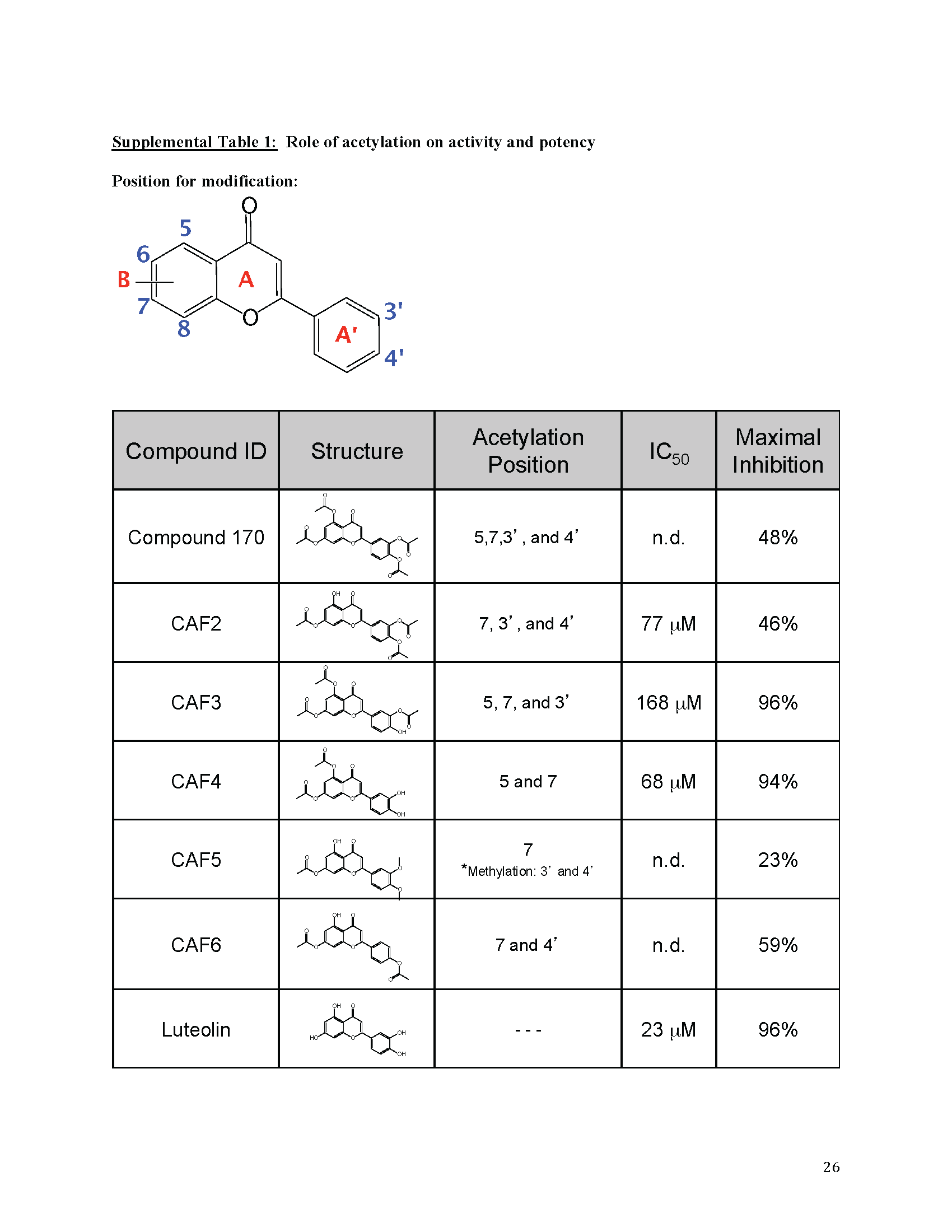

Supplement: Table S1 — Role of acetylation on activity and potency. (TIFF) [file pone.0084506.s003.tiff]

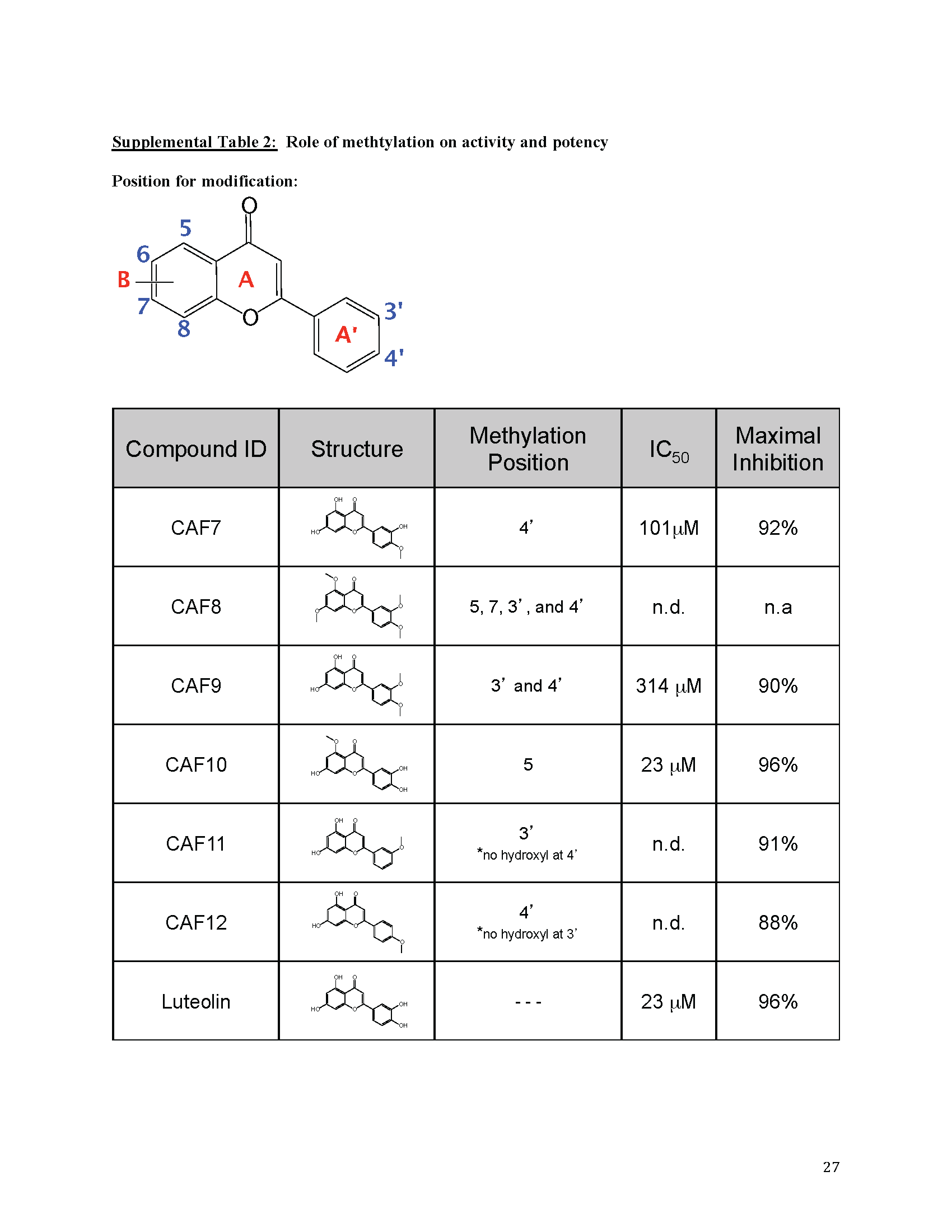

Supplement: Table S2 — Role of methylation on activity and potency. (TIFF) [file pone.0084506.s004.tiff]

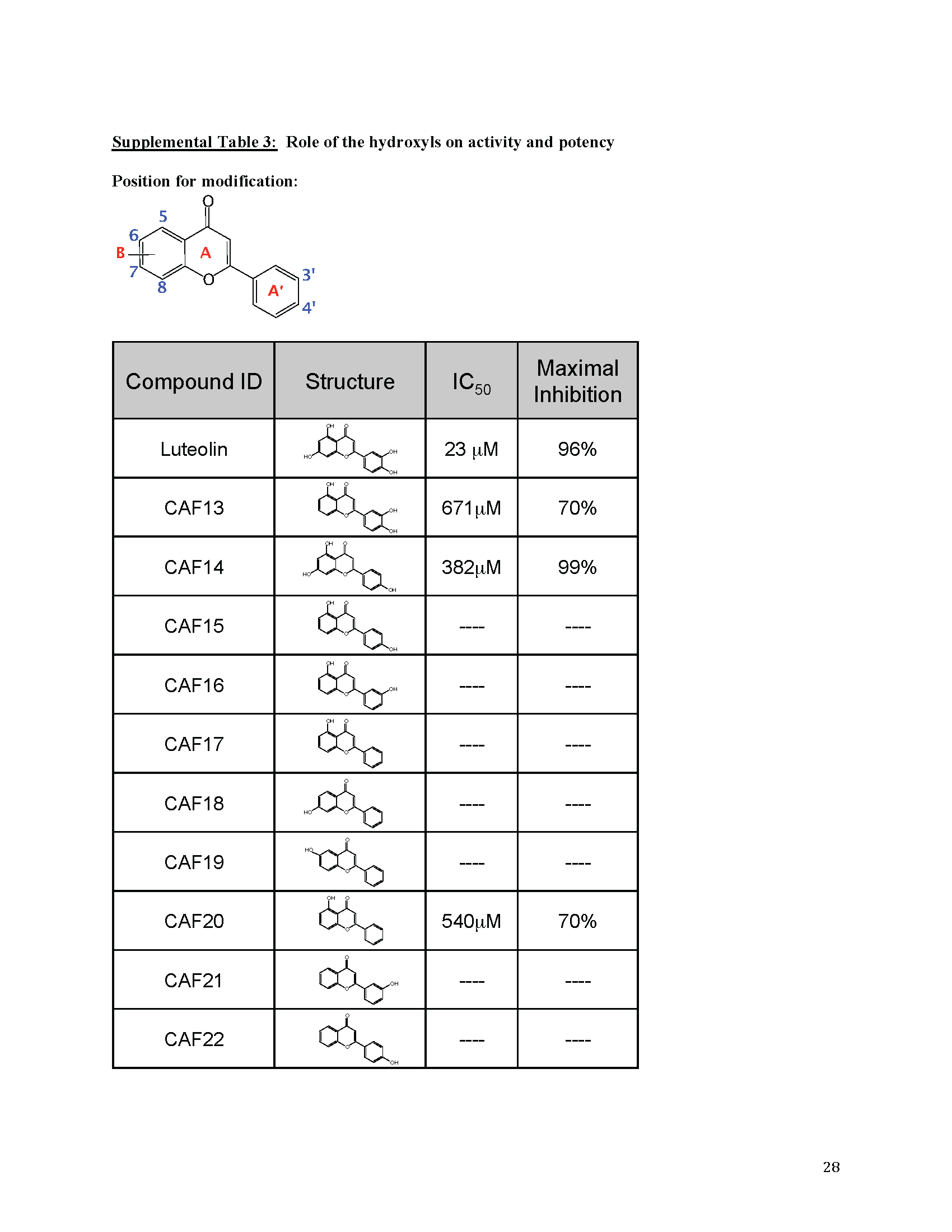

Supplement: Table S3 — Role of the hydroxyls on activity and potency. (TIFF) [file pone.0084506.s005.tiff]
